# *Toxoplasma gondii* affects trait anxiety in adult ADHD

**DOI:** 10.3389/fpsyt.2026.1766562

**Published:** 2026-03-05

**Authors:** Alexandra P. Lam, Angelika Carl, Klaus P. Kohse, Alexandra Philipsen

**Affiliations:** 1 Department of Psychiatry and Psychotherapy, University Hospital Bonn, University of Bonn, Bonn, Germany; 2 School of Medicine and Health Sciences, University of Oldenburg, Oldenburg, Germany; 3 University Institute of Clinical Chemistry and Laboratory Medicine, Klinikum Oldenburg, Oldenburg, Germany; 4 Institute for Laboratory Diagnostics and Microbiology, Klinikum Oldenburg, School of Medicine and Health Sciences, Carl von Ossietzky University of Oldenburg, Oldenburg, Germany

**Keywords:** ADHD, anxiety disorders, state anxiety, state-trait-anxiety inventory, *Toxoplasma gondii*, trait anxiety

## Abstract

**Background/objective:**

Growing evidence emerges that *Toxoplasma gondii* (*T. gondii*) is associated with mental disorders like anxiety disorders or attention-deficit/hyperactivity disorder (ADHD). In ADHD patients around 25% suffer from comorbid anxiety disorders. As the impact of a latent *T. gondii* infection on anxiety in adult ADHD remains unknown, this study aims to investigate this relationship.

**Methods:**

In a case-control study, including 140 participants, venous blood samples were taken of 70 adult ADHD patients and 70 controls for serological analysis of markers of infection and inflammation (leukocytes, C-reactive protein, anti-*T. gondii* immunoglobulin M (IgM) and anti-*T. gondii* immunoglobulin G (IgG) (seropositivity), IgG titers (serointensity) as well as anti-*T. gondii* IgG avidity. The influences on state and trait anxiety were explored using the State-Trait-Anxiety Inventory (STAI).

**Results:**

Seropositivity was significantly associated with the leukocyte count in all participants (*n* = 140, *p* = 0.004). Moreover, regression analysis revealed a significant association of seropositivity and serointensity with trait anxiety but not with state anxiety: trait anxiety was significantly lower in seropositive ADHD patients compared to seronegative subjects with ADHD (*n* = 70, p=0.029). In addition, trait anxiety scores decreased in an IgG-dependent manner in all participants (*n* = 140, *p* = 0.028) as well as in the ADHD group (*n* = 70, *p* = 0.015). Comorbid anxiety disorders in ADHD were not associated with latent *T. gondii* infection.

**Conclusion:**

Our data is the first revealing an association between *T. gondii* and trait anxiety in a serointensity-dependent manner in individuals with ADHD. Further research is needed to clarify the clinical impact of the observed lower trait anxiety in individuals with ADHD and latent *T. gondii* infection.

## Introduction

The obligate intracellular parasite *Toxoplasma gondii* (*T. gondii*) shows a high affinity for brain tissue and a potential life-long persistence, causing latent infections in the brain (1, 2). In humans, a growing number of studies suggests that *T. gondii* infection is associated with an increased risk of mental disorders like autism, schizophrenia, attention-deficit/hyperactivity disorder (ADHD), obsessive compulsive disorder, antisocial personality disorder, learning disabilities, and anxiety disorder (3–6) by immune activation, inflammatory mediators, or a direct influence on the nervous system (7, 8). However, data on the influence of latent *T. gondii* infection on anxiety are inconsistent, as increased as well as reduced anxiety-like symptoms in infected rats and mice have been reported (9, 10). In animal models, a reduced anxiety with an impaired mechanism of warning was found (11) while aversion against feline odor was found decreased or unchanged (9). Reduced fear toward cats displayed by infected rodents is associated with risky behavior and is discussed to increase the parasite’s chance of being transmitted to its definite host (2, 12).

A previous clinical study, conducted with patients suffering from diverse psychiatric disorders, found no association of *T. gondii* seropositivity neither with trait anxiety nor state anxiety (13). On the contrary, the prevalence of *T. gondii* was shown to be significantly associated with adult attention-deficit/hyperactivity disorder (ADHD) (6, 14) and latent *T. gondii* infection has been demonstrated to aggravate ADHD symptom severity (14). ADHD is a frequent neurodevelopmental disorder (15) showing a strong association with a wide range of psychiatric comorbidities (16). Around 80% of adults with ADHD suffer from at least one psychiatric comorbid disorder (17, 18). Anxiety disorders are among the most common co-occurring psychiatric diseases, with a comorbidity rate of around 25% (17). Individuals with ADHD and comorbid anxiety often show more severe anxiety symptoms with earlier onset of anxiety and more frequent additional comorbid psychiatric diagnoses than subjects without ADHD (17). However, the relationship of *T. gondii* on anxiety in ADHD remains unknown. This study aims to elucidate the impact of *T. gondii* on comorbid anxiety disorders as well as state and trait anxiety in ADHD patients and controls by investigating several serological markers of infection and inflammation of the humoral immune system (19).

## Materials and methods

### Recruitment and study sample

As part of a case-control study about the association between *T. gondii* and psychiatric diseases, patients of this clinic-based case-control study were consecutively recruited from the outpatient and inpatient units of the University Hospital of Psychiatry and Psychotherapy at the Carl von Ossietzky University of Oldenburg, School of Medicine and Health Sciences, Oldenburg (Germany) between May 2016 and November 2017. Controls were recruited via announcements at the website of the same university, without mentioning *T. gondii* in order to avoid over-selection of groups of people who are especially familiar with the topic or may have a particular interest in an antibody test against *T. gondii* (e.g. pregnant women, cat owners, et cetera). Laboratory analyses were performed at the Institute for Laboratory Diagnostics and Microbiology at the Klinikum Oldenburg and at the Medical Laboratory in Bremen. All methods were applied in accordance with the relevant guidelines and regulations. The study was approved by the local ethics committee (School of Medicine, University of Oldenburg, 2016-009). Written informed consent was obtained from all subjects before study participation. Patients were included if they fulfilled the criteria for ADHD according to DSM-IV. Every participant received 10 Euros as monetary compensation.

All study participants took part in the complete diagnostic process, comprising of diagnostic interviews and self-rating scales in German. The diagnosis (and exclusion) of ADHD in adulthood and other psychiatric disorders were established by psychiatric experts. ADHD in adulthood was established and validated using instruments such as the Wender-Utah-Rating-Scale for the retrospective assessment of ADHD in childhood (WURS-K) (20) and the ADHD Self-Rating Scale (ADHD-SR, German Version) (21). Severity of current ADHD-symptoms was assessed via the self-rated Conners’ Adult ADHD Rating Scale (CARRS:S-L, long version (22–24)), providing a balanced assessment of adult ADHD symptoms in different areas of life and indicating more severe symptoms by higher values. Further psychiatric disorders were assessed (or excluded) by using the structured clinical interview for DSM-IV (SCID-I, SCID-II; covering axis-I and personality disorders (25)), the Beck Depression Inventory (BDI-II (26), revised version 1996), and a self-rating form to assess autistic symptoms (Autismus Spektrum Quotienten (AQ (27)).

According to the ethic committee requirements of this study and to ensure valid diagnostic assessments, medication had not been discontinued abruptly prior to study begin. Termination of medication had been monitored by the treating physicians of the outpatient clinic. Medication was reduced stepwise over several weeks, until a minimal dosage had been reached, which could finally be terminated three days prior to study participation to limit potential withdrawal effects.

### Inclusion and exclusion criteria

Participants, at least 18 years old, who spoke and understood German, were included if no clinically significant abnormalities were detected on blood samples or physical examination. Patients were required to fulfill the criteria for ADHD according to DSM-IV. Exclusion criteria for patients and controls comprised of unwillingness or incapability to adhere to the study protocol, acute severe infection or inflammation, detected via CRP (excluded ≥5 mg/dl) and differential blood count, treatment with stimulants or ADHD-specific medication that cannot be terminated three days prior to blood sampling and questionnaires, severe abnormality known or detected on routine blood testing (i.e., thyroid dysfunction), pregnancy or breastfeeding. Moreover, controls were excluded if they had any psychiatric disorder except tobacco dependency or specific phobia, as no problematic relationship between these disorders and *T. gondii* is assumed.

### Questionnaire for anxiety strength

To evaluate strength of anxiety the State-Trait-Anxiety Inventory, the German version (STAI (28)) was used. The State-Trait-Anxiety Inventory (STAI) is a 40‐item self-rating instrument consisting of two subscales to measure state and trait anxiety. Twenty items relate to current symptoms of anxiety while 20 items relate to general, ongoing (trait) symptoms of anxiety. All items use a 4‐point Likert-scale to be answered by ‘almost never’, ‘sometimes’, ‘often’, and ‘almost always’. Scores range from 20 to 80. Higher scores represent higher levels of state and trait anxiety (28).

### Statistical analysis

Statistical analyses were conducted with SPSS Version 25. Descriptive analysis was performed for all variables included in the analyses. Frequencies were reported for categorical variables. Continuous variables were presented as mean and standard deviation if they have not earlier been published (14). To examine whether *T. gondii* seropositivity has an impact on comorbid anxiety disorders multiple logistic regression models were performed and Wald test was used to test for significance. These results are presented as ORs with 95% CIs. To examine whether *T. gondii* seropositivity has an impact on trait anxiety or state anxiety, multiple linear regression models were constructed with *T. gondii* seropositivity (ref ‘no’). All regression models were estimated for the entire study sample and for the ADHD group. In secondary analyses, the associations between IgG or anti-*T. gondii* IgG avidity (measured as share of affinity) and anxiety disorders, state anxiety as well as trait anxiety were estimated. For each regression model, a separate stepwise backward variable elimination was performed. In the variable selection process with regard to anxiety disorders, state or trait anxiety as dependent variables, the covariates were set as follows: age (in years), sex (ref ‘female’), IgG (U/ml) or *T. gondii* seropositivity (ref ‘no’), ADHD (ref ‘no’), borderline personality disorder (BPD) as the most frequent Axis II disorder in this sample (ref ‘no’), Axis II other than BPD (ref ‘no’), affective disorders (ref ‘no’), anxiety disorder (ref ‘no’) if anxiety disorder was not the dependent variable, lifetime substance abuse (ref ‘no’), eating disorder (ref ‘no’), antipsychotic medication (ref ‘no’), antidepressant medication (ref ‘no’), sedatives (ref ‘no’), and ADHD medication (ref ‘no’). Linear regression analysis results were reported as regression coefficients with 95% confidence intervals (CI). Scatter plots were fitted by bivariate linear regression of the whole study sample and the ADHD group.

Linear regression analyses were performed to examine the impact of *T. gondii* on the leukocyte count in ADHD patients and controls. In the variable selection process with regard to the level of leukocytes the covariates were set as follows: substance abuse or dependence except smoking (ref ‘no’), *T. gondii* positive (ref ‘no’), level of CRP, current pharmacological ADHD treatment (ref ‘no’), anxiety disorder (ref ‘no’), number of smoked cigarettes (ref ‘no’), age, hypnotics or sedatives (ref ‘no’), sex (ref ‘female’), antidepressants (ref ‘no’), neuroleptics (ref ‘no’), affective disorders (ref ‘no’), eating disorder (ref ‘no’). Linear regression analysis results were reported as regression coefficients with 95% CIs. Means of trait and state anxiety scores between groups were compared by unpaired *t*-test. The statistical significance level was set to *p* < 0.05.

### Laboratory analysis

Venous blood samples were taken from all eligible participants in the morning between 8:00 and 9:00 am. ADHD medication had to be discontinued at least three days prior to the diagnostic assessment. As previously described, the enzyme immunoassay kit Enzygnost^®^ Toxoplasmosis IgG and IgM (Siemens Healthcare Diagnostics Products GmbH, Marburg, Germany) was used to test all blood samples for *T. gondii* IgG and IgM antibodies (29). Serological assays were performed on the automated system BEP 2000^®^ (Siemens Healthcare Diagnostics Products GmbH, Marburg, Germany). According to the instructions of the manufacturer, *T. gondii* antibody titers were categorized as negative (< 6 U/ml) or positive (> 6 U/ml). Additionally, a sub-analysis of the serointensity was performed, as previously described (14), in seropositive respondents and concentrations of anti-*T. gondii* IgG were obtained in units per milliliter (U/ml). Moreover, the avidity of *T. gondii* antibodies was tested by using the fully automated chemiluminescence analyzer LIAISON^®^ XL (DiaSorin S.p.A. Via Crescentino, snc, Saluggia (30), Italy). The avidity index allows specimen classification as low (avidity index, <0.2), moderate (avidity index, 0.20 to 0.30), or high (avidity index, >0.3) avidity. In addition, an immunoturbidimetric assay was used to determine the CRP concentrations on a cobas 6000 analyzer system (Roche Diagnostics GmbH, Germany). The Advia 2120-System^®^ (Siemens Healthcare Diagnostics) was used to perform differential blood counts. If necessary, the serum samples were stored at -80 °C.

## Results

### Characteristics and comorbid anxiety disorder

In total, the study sample comprises 140 participants, with *n* = 70 patients with adult ADHD and *n* = 70 control subjects. Both groups were balanced with regard to age and sex. Twenty-eight participants (20.0%) were seropositive for anti-*T. gondii* immunoglobulin G (IgG), and zero participants were seropositive for anti-*T. gondii* immunoglobulin M ( 14, 31). As part of a larger case-control study about the association between *T. gondii* and psychiatric diseases, demographic and screening characteristics of the study sample have been described in detail earlier (14). As previously published, significantly more individuals were found seropositive for anti-*T. gondii* IgG in the ADHD group (*n* = 19, 27.1%) compared to (*n* = 9, 12.9%) in the control group (χ^2^ = 4.46; *n* = 140; *p* = 0.035) (14). Regarding comorbid disorders, 62.9% of ADHD patients suffered from at least one current comorbid Axis I and 31.4% of at least one current comorbid Axis II disorder (14). With regard to anxiety, *n* = 14 (20%) in the ADHD group had comorbid anxiety disorders: *n* = 2 (2.9%) social phobia, *n* = 4 (5.7%) posttraumatic stress disorder (PTSD, *n* = 4 (5.7%) panic disorder, *n* = 3 (4.3%) specific phobia, *n* = 1 (1.4%) general anxiety disorder. In seropositive patients with ADHD, *n* = 10 (19.6%) had at least one comorbid anxiety disorder while *n* = 41 (80.4%) did not have any comorbid anxiety disorder. N = 4 (21.1%) seronegative patients were diagnosed with at least one anxiety disorder, while *n* = 15 (79.0%) did not have anxiety disorders. In the controls, n=2 (2.9%) reported specific phobia like arachnophobia, which represented no exclusion criteria. Both controls were seronegative for anti-*T. gondii* IgG. Multiple variable analyses of the whole study population found no association between anxiety disorders and *T. gondii* seropositivity, serointensity or avidity in our study sample. Consequently, participants with anxiety disorders were not excluded in further analyses. However, significant associations between comorbid anxiety disorders were found for age (Odds Ratio (OR) 1.069, 95% CI 1.007-1.134, *p* = 0.028) and female sex (OR 0.220, 95% CI 0.055-0.874, *p* = 0.031) in the ADHD group. Regarding the entire study sample, age (OR 1.065, 95% CI 1.008-1.125, *p* = 0.026), female sex (OR 0.182, 95%CI 0.049-0.685, *p* = 0.012), and ADHD (OR 8.208, 95% CI 1.725-39.047, *p* = 0.008), showed significant associations with anxiety disorders in logistic regression analyses.

ADHD patients revealed significantly elevated scores with regard to state anxiety (*n* = 70, mean 49.79, SD = 8.835, min. 33, max. 69) compared to controls (*n* = 70, mean 34.90, SD = 7.392, min. 20, max. 56), t (138)=-10.815, *p* < 0.001. Trait anxiety scores were also significantly increased in the ADHD group (*n* = 69, mean 51.93, SD = 9.772, min 34, max 72) compared to the control group (*n* = 70, mean 35.06, SD = 8.095, min 20, max 58), t (138)=-11.124, *p* < 0.001.

As previously published, 27.1% in the ADHD group of the study sample (*n* = 19) received antidepressants, 8.6% narcoleptics (*n* = 6), 4.3% sedatives (*n* = 3), and 30% took medication for physical alignments (*n* = 21) (14). Of all participating ADHD patients (*n* = 70), the majority was treated by ADHD medications, while 47.1% (*n* = 33) did not take any ADHD medication. Of those, who received ADHD medication prior to study participation, 88.9% (*n* = 32) took long-acting methylphenidate, 2.7% (*n* = 1) took lisdexamfetamine, and 16.2% (*n* = 6) atomoxetine.

### Leukocytes and *T. gondii*

The mean C-reactive protein concentration (CRP) was 0.04 mg/dl (± 0.26) in *n* = 140 participants, the leukocyte count 6,360/µl (± 1,800) measured in *n* = 140 participants. Multiple regression analysis revealed a significant influence of *T. gondii* seropositivity on the leukocyte count (B = 1.745; 95% CI 0.592-2.898 *p* = 0.004). In addition to *T. gondii*, the CRP level (B = 1.857; 95% CI 0.339-3.374, *p* = 0.018) and the number of smoked cigarettes (B = 0.121; 95% CI 0.052-0.191, *p* = 0.001) showed a significant effect on the leukocyte count.

### Seropositivity and anxiety

Multiple linear regression analysis revealed a significant association between *T. gondii* seropositivity and trait anxiety with regard to the entire sample (see **Table 1a**) and individuals with ADHD (see **Supplementary Table 1a**).

**Table 1a:** Multiple linear regression model of *T. gondii* seropositivity and trait anxiety in all cases.

Trait anxiety, all cases							
First model: included variables (*n* = 140, adjusted R^2^ = 0.551, *p* < 0.001)							
						95% CI	
	B	SE B	*β*	T	*p*	Lower	Upper
**(Intercept)**	34.632	2.455		14.108	0.000	29.774	39.491
Age	-0.001	0,077	-0.001	-0.011	0.992	-0.153	0.151
Sex	1.413	1.541	0.058	0.917	0.361	-1.636	4.463
*T. gondii* seropositivity	-3.490	1.836	-0.114	-1.901	0.060	-7.124	0.144
ADHD	14.008	2.444	0.573	5.731	**0.000**	9.171	18.845
BPD	8.277	3.274	0.203	2.528	**0.013**	1.797	14.757
Axis II disorder other than BPD	8.303	3.434	0.158	2.418	**0.017**	1.506	15.100
Affective disorder	4.683	2.154	0.163	2.174	**0.032**	0.419	8.947
Anxiety disorder	2.838	2.525	0.074	1.124	0.263	-2.159	7.836
Substance abuse lifetime	-0.758	2.664	-0.021	-0.285	0.776	-6.030	4.514
Eating disorder	-5.993	5.447	-0.071	-1.100	0.273	-16.773	4.787
Neuroleptics	0.752	4.492	0.012	0.167	0.867	-8.138	9.642
Antidepressants	-0.396	2.876	-0.011	-0.138	0.891	-6.089	5.296
Sedatives	2.078	6.233	0.025	0.333	0.739	-10.258	14.415
Current pharmacological ADHD treatment	-2.549	2.397	-0.092	-1.063	0.290	-7.294	2.195

Final model: included variables (*n* = 140, adjusted R^2^ = 0.558, *p* < 0.001)							
						95% CI	
	B	SE B	*β*	T	*p*	Lower	Upper
**(Intercept)**	35.374	0.993		35.620	**0.000**	33.409	37.338
*T. gondii* seropositivity	-3.462	1.742	-0.113	-1.988	**0.049**	-6.907	-0.018
ADHD	12.637	1.643	0.517	7.690	**0.000**	9.387	15.887
BPD	8.867	2.567	0.218	3.454	**0.001**	3.790	13.944
Axis II disorder other than BPD	8.656	3.143	0.164	2.754	**0.007**	2.440	14.873
Affective disorder	4.610	1.942	0.160	2.374	**0.019**	0.770	8.450

Final model: excluded variables							
			β In	T	*p*	Partial Correlation	Collinearity Statistics Tolerance
Age			0.016	0.267	0.790	0.023	0.904
Antidepressants			-0.012	-0.170	0.865	-0.015	0.605
Neuroleptics			0.004	0.058	0.954	0.005	0.719
Substance abuse lifetime			0.025	0.399	0.690	0.035	0.786
Sedatives			-0.011	-0.175	0.861	-0.015	0.854
Sex			0.053	0.923	0.358	0.080	0.952
Anxiety disorder			0.057	0.964	0.337	0.083	0.908
Eating disorder			-0.062	-1.080	0.282	-0.093	0.961
Current pharmacological ADHD treatment			-0.082	-1.134	0.259	-0.098	0.598

B, unstandardized coefficient**;** β, standardized coefficient; *p*, p-value; CI, 95% confidence interval for [B]; *T. gondii*, *Toxoplasma gondii*; ADHD, attention-deficit/hyperactivity disorder; BPD, borderline personality disorder; bold, significant result (*p* < 0.05). “No” is the reference category for all categorical variables.

**Figure 1:**
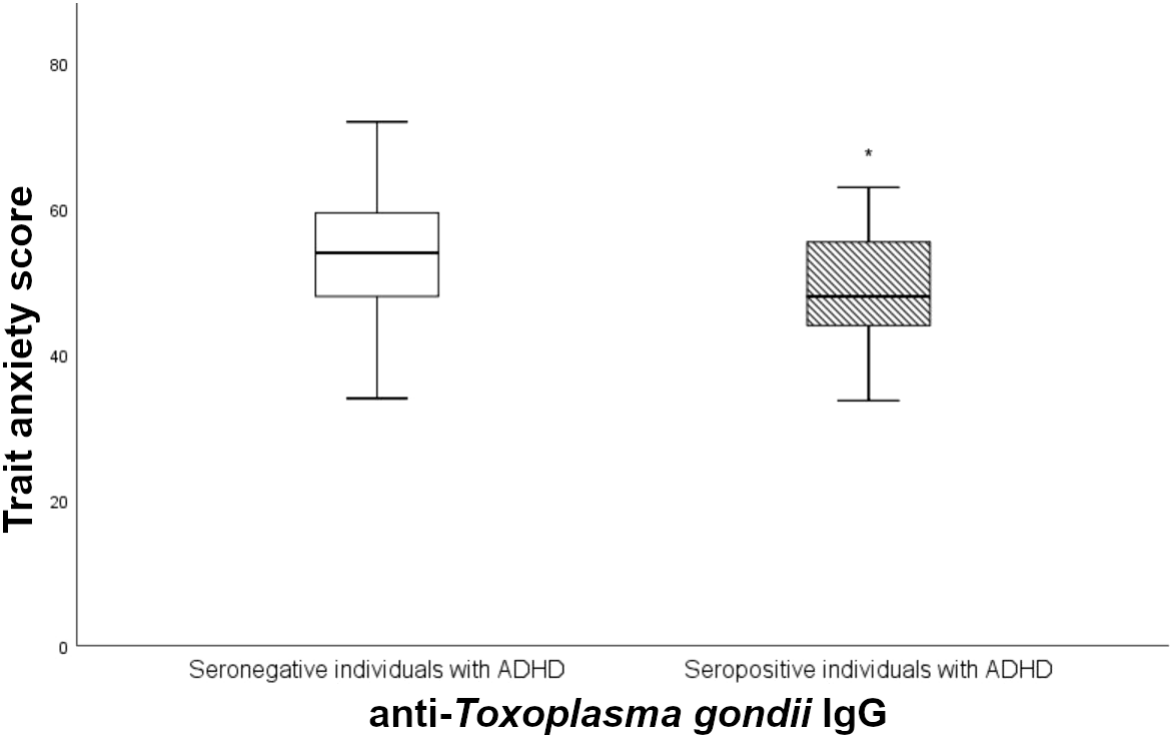
Mean trait anxiety scores in anti-*T. gondii* IgG seropositive and seronegative individuals with ADHD, presented inbox plots of the ADHD group (*n* = 70). The lower and upper box boundaries show the 25th and 75th percentiles, respectively; the horizontal line inside box depicts the median; the box contains the middle 50% of recorded data; the error bars display the minimum and maximum values; *, significant influence of *T. gondii* found in linear regression models; IgG, immunoglobulin G.

*T. gondii* seropositive ADHD patients showed significantly reduced trait anxiety scores as compared to *T. gondii* seronegative individuals with ADHD t (68)=1.936, *p* = 0.029 (see **Figure 1**). However, in the controls, no significant difference could be found between seropositive and seronegative individuals t (68)=0.243, *p* = 0.405. Additionally, multiple linear regression analyses revealed no significant association between *T. gondii* seropositivity and state anxiety (see **Table 2b**; **Supplementary Table 1b**).

**Table 1b:** Multiple linear regression model of *T. gondii* seropositivity and state anxiety in all cases.

State anxiety, all cases							
First model: included variables (*n* = 140, adjusted R^2^ = 0.481, *p* < 0.001)							
						95% CI	
	B	SE B	*β*	T	*p*	Lower	Upper
**(Intercept)**	34.105	2.373		14.373	**0.000**	29.409	38.802
Age	0.024	0.074	0.023	0.328	0.743	-0.123	0.171
Sex	0.959	1.489	0.044	0.644	0.521	-1.988	3.907
*T. gondii* seropositivity	-3.325	1.775	-0.121	-1.873	0.063	-6.838	0.188
ADHD	13.296	2.363	0.605	5.628	**0.000**	8.620	17.972
BPD	9.717	3.165	0.265	3.070	**0.003**	3.453	15.980
Axis II disorder other than BPD	9.423	3.320	0.199	2.838	**0.005**	2.853	15.994
Affective disorder	1.832	2.082	0.071	0.880	0.381	-2.289	5.953
Anxiety disorder	-1.622	2.441	-0.047	-0.665	0.508	-6.453	3.209
Substance abuse lifetime	-2.771	2.575	-0.086	-1.076	0.284	-7.867	2.326
Eating disorder	0.620	5.265	0.008	0.118	0.906	-9.801	11.040
Neuroleptics	-5.611	4.342	-0.103	-1.292	0.199	-14.204	2.983
Antidepressants	-0.627	2.780	-0.020	-0.226	0.822	-6.130	4.875
Sedatives	-2.875	6.025	-0.038	-0.477	0.634	-14.800	9.050
Current pharmacological ADHD treatment	-0.019	2.317	-0.001	-0.008	0.993	-4.606	4.567

Final model: included variables (*n* = 140, adjusted R^2^ = 0.50, *p* < 0.001)							
						95% CI	
	B	SE B	*β*	T	*p*	Lower	Upper
**(Intercept)**	35.262	0.957		36.845	**0.000**	33.370	37.155
*T. gondii* seropositivity	-2.818	1.680	-0.103	-1.677	0.096	-6.140	0.505
ADHD	13.049	1.481	0.594	8.810	**0.000**	10.120	15.979
BPD	6.721	2.371	0.183	2.834	**0.005**	2.031	11.411
Axis II disorder other than BPD	7.870	2.984	0.166	2.637	**0.009**	1.969	13.772

Final model: excluded variables							
			β In	T	*p*	Partial correlation	Collinearity statistics tolerance
Current pharmacological ADHD treatment			0.031	0.394	0.694	0.034	0.600
Eating disorder			0.01	0.164	0.870	0.014	0.968
Antidepressants			-0.01	-0.120	0.905	-0.010	0.671
Age			0.03	0.477	0.634	0.041	0.908
Hypnotics. sedatives			-0.06	-0.901	0.369	-0.078	0.872
Anxiety disorder			-0.01	-0.190	0.849	-0.016	0.916
Affective disorder			0.028	0.392	0.695	0.034	0.687
Sex			0.04	0.652	0.515	0.056	0.952
Substance abuse lifetime			-0.07	-1.087	0.279	-0.093	0.787
Neuroleptics			-0.1	-1.463	0.146	-0.125	0.756

B, unstandardized coefficient**;** β, standardized coefficient; *p*, p-value; CI, 95% confidence interval for [B]; *T. gondii*, *Toxoplasma gondii*; ADHD, attention-deficit/hyperactivity disorder; BPD, borderline personality disorder; bold, significant result (*p* < 0.05). “No” is the reference category for all categorical variables.

### Serointensity and anxiety

To clarify to what extent trait or state anxiety were influenced by serointensity, further multiple linear regression models were applied. Trait anxiety was significantly affected by the anti-*T. gondii* IgG titer in the regression analyses. Multiple linear regression models revealed a significant negative association between anti-*T. gondii* IgG and trait anxiety scores in the entire study cohort (unstandardized coefficient (B) = - 0.38; 95%-CI -0.071–0.004, *p* = 0.028), (see **Table 2a**) as well as in the group of ADHD patients (see **Supplementary Table 2a**). Further, results of the stepwise multiple regression indicated that, in addition to anti-*T. gondii* IgG, ADHD, affective disorders, BPD, and other Axis II disorders had a significant influence on trait anxiety in the whole study population (see **Table 2a**). When combined, all the significant predictors in the final model accounted for 56.9% (R^2^ = 0.569, F = 37.63, *p* < 0.001) of the variance in trait anxiety scores of the STAI in the entire study population.

**Table 2a:** Multiple linear regression model of serointensity and trait anxiety in all cases.

Trait anxiety, all cases							
First model: included variables (*n* = 140, adjusted R^2^ = 0.553, *p* < 0.001)							
						95% CI	
	B	SE B	*β*	T	*p*	Lower	Upper
**(Intercept)**	34.814	2.441		14.261	**0.000**	29.982	39.646
Age	-0.011	0.076	-0.009	-0.148	0.883	-0.161	0.139
Sex	1.453	1.538	0.059	0.945	0.347	-1.590	4.496
IgG [U/ml]	-0.037	0.018	-0.123	-2.066	**0.041**	-0.072	-0.002
ADHD	14.335	2.457	0.587	5.835	**0.000**	9.474	19.197
BPD	7.627	3.283	0.187	2.323	**0.022**	1.129	14.124
Axis II disorder other than BPD	7.592	3.424	0.144	2.217	**0.028**	0.815	14.369
Affective disorder	4.425	2.152	0.154	2.057	**0.042**	0.167	8.684
Anxiety disorder	2.598	2.527	0.068	1.028	0.306	-2.404	7.600
Substance abuse lifetime	-0.939	2.658	-0.026	-0.353	0.725	-6.200	4.323
Eating disorder	-6.094	5.426	-0.072	-1.123	0.264	-16.832	4.645
Neuroleptics	0.898	4.480	0.015	0.200	0.841	-7.969	9.765
Antidepressants	-0.104	2.882	-0.003	-0.036	0.971	-5.807	5.600
Sedatives	2.521	6.185	0.030	0.408	0.684	-9.719	14.761
Current pharmacological ADHD treatment	-2.508	2.390	-0.091	-1.049	0.296	-7.237	2.221

Final model: included variables (*n* = 140, adjusted R^2^ = 0.569, *p* < 0.001)							
						95% CI	
	B	SE B	*β*	T	*p*	Lower	Upper
**(Intercept)**	35.259	0.976		36.143	**0.000**	33.330	37.189
IgG [U/ml]	-0.038	0.017	-0.127	-2.224	**0.028**	-0.071	-0.004
ADHD	12.936	1.657	0.529	7.808	**0.000**	9.659	16.213
BPD	8.376	2.575	0.206	3.252	**0.001**	3.282	13.469
Axis II disorder other than BPD	8.022	3.130	0.152	2.563	**0.011**	1.831	14.212
Affective disorder	4.416	1.935	0.153	2.282	**0.024**	0.589	8.243

Final model: excluded variables							
			β In	T	*p*	Partial correlation	Collinearity statistics tolerance
Antidepressants			-0.004	-0.053	0.958	-0.005	0.600
Age			0.009	0.149	0.882	0.013	0.925
Neuroleptics			0.01	0.149	0.882	0.013	0.722
Substance abuse lifetime			0.021	0.338	0.736	0.029	0.785
Sedatives			-0.003	-0.047	0.962	-0.004	0.863
Sex			0.053	0.930	0.354	0.080	0.954
Anxiety disorder			0.049	0.829	0.408	0.072	0.904
Eating disorder			-0.061	-1.070	0.286	-0.092	0.961
Current pharmacological ADHD treatment			-0.078	-1.078	0.283	-0.093	0.598

B, unstandardized coefficient**;** β, standardized coefficient; *p*, p-value; CI, 95% confidence interval for [B]; IgG, anti-*T. gondii* immunoglobulin G; ADHD, attention-deficit/hyperactivity disorder; BPD, borderline personality disorder; bold, significant result (*p* < 0.05). “No” is the reference category for all categorical variables.

**Figure 2:**
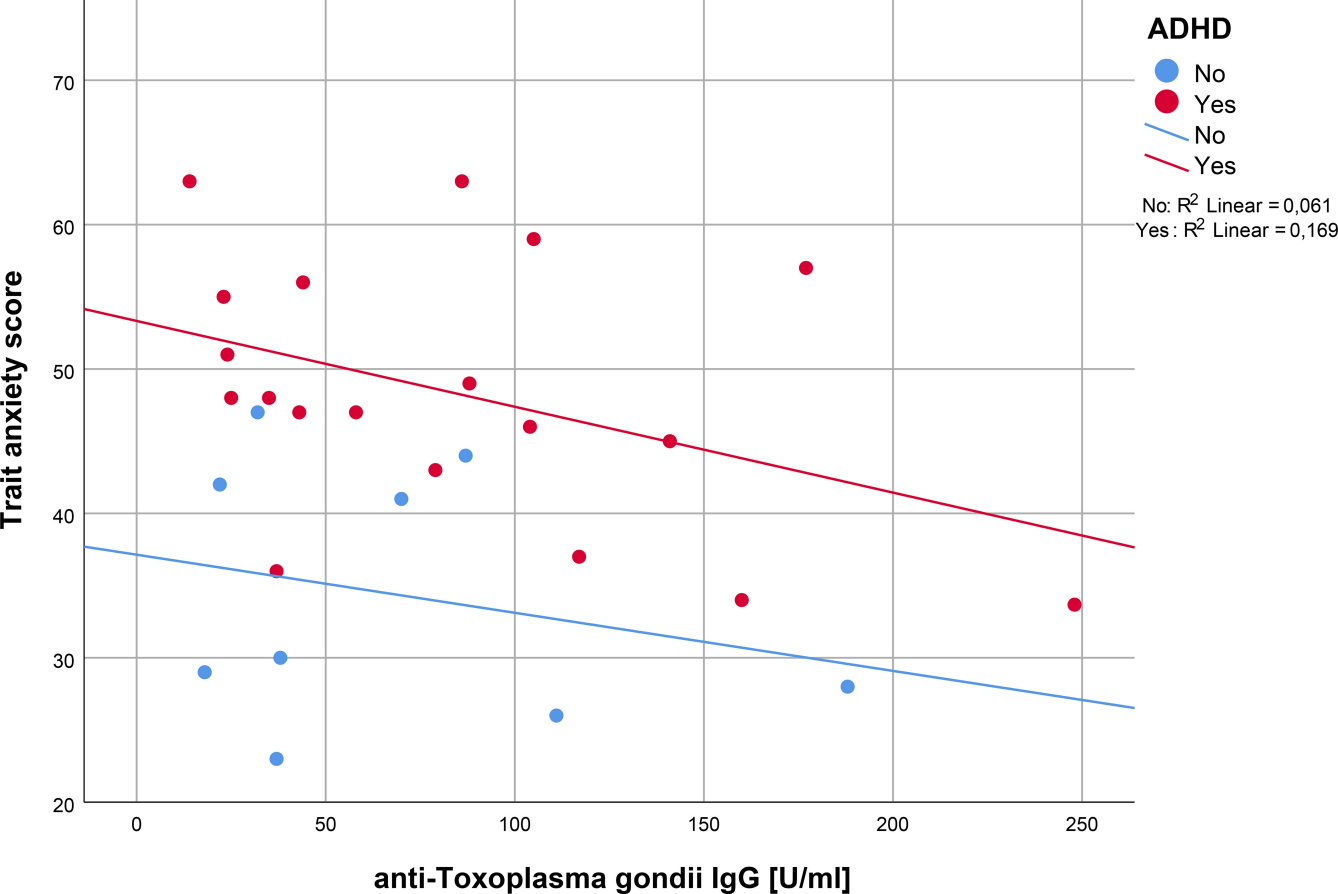
Regression analysis of anti-*T. gondii* IgG and trait anxiety in ADHD patients versus controls. IgG, immunoglobulin G; ADHD, attention-deficit/hyperactivity disorder.

**Figure 3:**
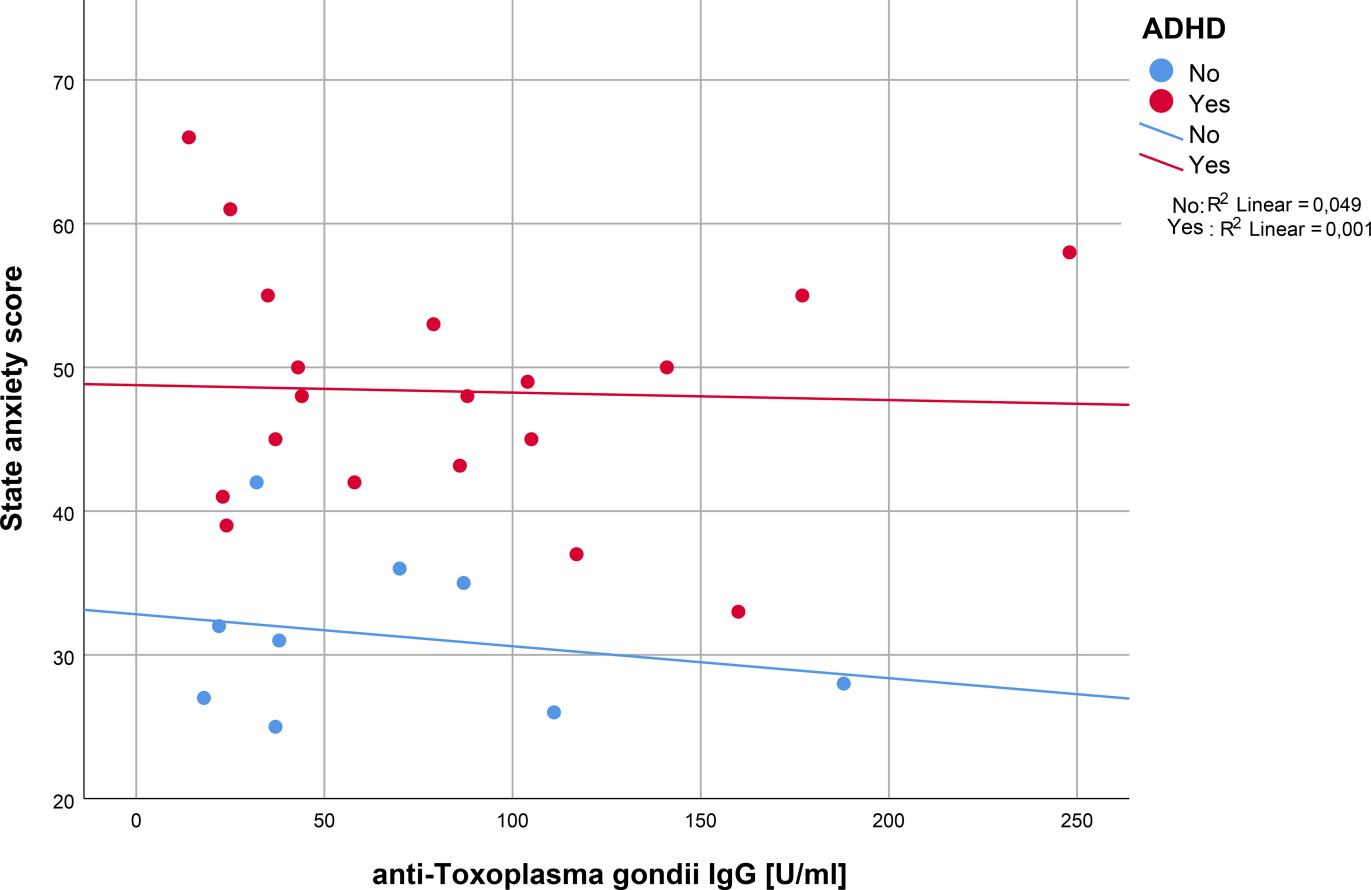
Regression analysis of anti-*T. gondii* IgG and state anxiety in ADHD patients versus controls. IgG, immunoglobulin G; ADHD, attention-deficit/hyperactivity disorder.

Spearman’s correlation analysis for trait anxiety and anti-*T. gondii* IgG serointensity revealed a significant result in ADHD patients, but not in controls (Spearman’s rho ADHD: -0.267, *p* = 0.026, Spearman’s rho controls: -0.267, *p* = 0.244), (see **Figure 2**). In contrast, the correlation analysis for state anxiety and serointensity did not reveal significant correlations, neither in ADHD patients (Spearman’s rho: -0.077, *p* = 0.377), nor in controls (Spearman’s rho: -0.083, *p* = 0.416), (see **Figure 3**).

However, multiple regression analysis revealed that having ADHD, BPD, or other Axis II disorders affects state anxiety significantly (see **Table 2b**; **Supplementary Table 2b**). In the final model, all those significant predictors accounted for 49.3% (R^2^ = 0.493, F = 46.116, *p* < 0.001) of the variance in state anxiety scores of the STAI in the entire study population (see **Table 2b**).

**Table 2b:** Multiple linear regression model of serointensity and state anxiety in all cases.

State anxiety, all cases							
First model: included variables (*n* = 140, adjusted R^2^ = 0.472, *p* < 0.001)							
						95% CI	
	B	SE B	*β*	T	*p*	Lower	Upper
**(Intercept)**	34.380	2.387		14.404	**0.000**	29.656	39.104
Age	0.008	0.074	0.007	0.104	0.918	-0.139	0.154
Sex	0.925	1.503	0.042	0.615	0.540	-2.051	3.900
IgG [U/ml]	-0.019	0.017	-0.073	-1.120	0.265	-0.054	0.015
ADHD	13.258	2.402	0.603	5.520	**0.000**	8.504	18.011
BPD	9.393	3.210	0.256	2.926	**0.004**	3.040	15.746
Axis II disorder other than BPD	8.891	3.348	0.188	2.655	**0.009**	2.264	15.517
Affective disorder	1.684	2.104	0.065	0.801	0.425	-2.479	5.848
Anxiety disorder	-1.624	2.471	-0.047	-0.657	0.512	-6.515	3.266
Substance abuse lifetime	-2.872	2.599	-0.089	-1.105	0.271	-8.016	2.272
Eating disorder	0.171	5.305	0.002	0.032	0.974	-10.329	10.670
Neuroleptics	-5.485	4.380	-0.101	-1.252	0.213	-14.154	3.185
Antidepressants	-0.665	2.818	-0.021	-0.236	0.814	-6.241	4.912
Sedatives	-1.943	6.047	-0.026	-0.321	0.748	-13.911	10.024
Current pharmacological ADHD treatment	0.115	2.336	0.005	0.049	0.961	-4.509	4.738

Final model: included variables (*n* = 140, adjusted R^2^ = 0.493, *p* < 0.001)							
						95% CI	
	B	SE B	*β*	T	*p*	Lower	Upper
**(Intercept)**	34.900	0.939		37.186	**0.000**	33.044	36.756
ADHD	12.648	1.472	0.575	8.595	**0.000**	9.738	15.558
BPD	6.880	2.385	0.188	2.885	**0.005**	2.164	11.597
Axis II disorder other than BPD	7.577	2.999	0.160	2.527	**0.013**	1.647	13.507

Final model: excluded variables							
			β In	T	*p*	Partial correlation	Collinearity statistics tolerance
Eating disorder			0.007	0.111	0.912	0.010	0.969
Current pharmacological ADHD treatment			0.032	0.410	0.683	0.035	0.600
Age			0.009	0.150	0.881	0.013	0.942
Antidepressants			-0.017	-0.232	0.817	-0.020	0.674
Sedatives			-0.046	-0.710	0.479	-0.061	0.882
Anxiety disorder			-0.012	-0.196	0.845	-0.017	0.916
Affective disorder			0.026	0.356	0.722	0.031	0.688
Sex			0.029	0.472	0.638	0.041	0.963
Substance abuse lifetime			-0.074	-1.084	0.280	-0.093	0.787
IgG [U/ml]			-0.062	-1.003	0.318	-0.086	0.950
Neuroleptics			-0.093	-1.344	0.181	-0.115	0.759

B, unstandardized coefficient**;** β, standardized coefficient; *p*, p-value; CI, 95% confidence interval for [B]; IgG, anti-*T. gondii* immunoglobulin G; ADHD, attention-deficit/hyperactivity disorder; BPD, borderline personality disorder; bold, significant result (*p* < 0.05). “No” is the reference category for all categorical variables.

### Anxiety and avidity

Multiple regression analyses revealed no statistical association between anti-*T. gondii* IgG avidity and state anxiety or trait anxiety, neither in ADHD patients nor in controls.

## Discussion

While humans are dead-end hosts for *T. gondii*, latent infection with the parasite, however, is one of the most common and apart from congenital transmission generally was considered as asymptomatic (32). This assumption has been reappraised, as latent *T. gondii* infections have shown behavioral alterations in rodents and other mammal models (32). Although humans are accidental hosts of the parasite and *T. gondii* infection in humans are unlikely to reflect adaptive host manipulation, broad effects of the parasite on human behavior has been intensively studied and discussed (32, 33). However, studies investigating the relationship between *T. gondii* and anxiety in humans are limited and mainly focused on the association of seropositivity and anxiety disorders (34). In a recent meta-analysis, Dowran et al. found eight human studies indicating a relationship between *T. gondii* and anxiety, and three studies without significant association between *T. gondii* and anxiety disorders (34). Available studies evaluating the levels of anxiety in *T. gondii* seropositive psychiatric patients are even scarcer. Coccaro et al. investigated the association of *T. gondii* with categorical and dimensional measures of aggression using the STAI in patients with intermittent explosive disorder (IED), non-IED psychiatric disorders (psychiatric controls), or no evidence of any psychiatric diagnosis (healthy controls) and found no effect neither on state nor trait anxiety (13).

None of the mentioned studies included patients with ADHD. As far as we are aware, the association between *T. gondii* and anxiety in ADHD has not been investigated yet. In addition, the case-control study presented here is the first to explore the impact of *T. gondii* seropositivity on the level of anxiety in ADHD patients and healthy controls. In line with results of a former meta-analysis (35), anxiety disorders were significantly associated with age and female sex in our study. Despite that, no association of a latent *T. gondii* infection and comorbid anxiety disorders could be shown for the study population. To our knowledge, our results are the first describing a significantly lower trait anxiety in adult ADHD patients, dependent of *T. gondii* seropositivity and IgG serointensity.

In adults with ADHD, the STAI questionnaires are often used to assess anxiety levels (36). *“State anxiety”* is defined as a temporary reaction or intense emotional state to adverse events (37). In contrast, “*trait anxiety*” is regarded as a more stable personality feature, a tendency to respond to troubles, concerns, and worries in different situations (37). In line with our findings, previous studies in adults with ADHD found higher levels of trait anxiety than state anxiety as well as higher levels of anxiety compared to controls (38–40). In further studies, it has been found that trait anxiety can predict students’ performance in attention tasks (41) and that showing anxiety has a strong impact on functional impairment in ADHD (42), whereas state anxiety demonstrated significant explanatory power over attentional variables in a virtual reality continuous performance test (43). Moreover, differences with regard to the subtype of ADHD have been described. While the combined subtype was reported to reveal higher trait anxiety, the inattentive subtype demonstrated higher state anxiety (41). Adult psychiatric outpatients with depressive or anxiety disorders and comorbid ADHD showed significantly higher state and trait anxiety scores than non-ADHD patients (38). In our study presented here, significantly elevated mean state and trait anxiety scores were also found in individuals with ADHD when compared to controls. However, it is striking that ADHD patients revealed anti-*T. gondii* IgG dependent reduced trait anxiety scores compared to seronegative individuals with ADHD.

### Discussed parasite-driven alterations

Although the underlying neurobiological mechanisms remain hypothetical and unproven, our findings suggest a possible combined alteration of the affected dopaminergic system through ADHD and a latent *T. gondii* infection, which clinically might contribute to a decrease of trait anxiety and has not been reported before. Even though speculative, this particular reduction of the trait anxiety could be assumed as an anxiolytic effect of *T. gondii* on individuals with ADHD.

In mammals, anxiolytic behaviors after *T. gondii* infection have been described (44). Although direct human-specific evidence for parasite-driven neural alterations is lacking (45), several studies suggest an influence of *T. gondii* on behavioral changes through a range of mechanisms like inflammatory mediators and immune response, the modulation of neurotransmitters, hormones, as well as a direct influence on the nervous system by interference with the neurons and brain regions that mediate behavioral expression (7, 8, 46). The amygdala was particularly found to be one of the brain regions consistently more infected by cysts of *T. gondii* than others (1, 47). In concordance, the potential influence of *T. gondii* on dopaminergic signaling augmentation in the amygdala is discussed to activate the anxiety circuitry inappropriately (48). Corticosterone, acting within the basolateral amygdala, has been shown to enhance the fear response to environmental stimuli, like aversive cat odor, in rats (44). *T. gondii* infection was found to effect this fear response by inducing dendritic retraction in the basolateral amygdala associated with an non-physiological reduced corticosterone secretion in rats (44). Especially, the predominant *T. gondii* cyst affinity to limbic areas is proposed to work as a natural anxiolytic mechanism (11).

Limbic regions such as the amygdala and cingulate cortices are functionally and structurally involved in anxiety disorders, and trait anxiety, in particular, can be regarded as risk factor for mood and anxiety disorders (49, 50). Regarding state and trait anxiety alterations in general, amygdala activation is especially thought to play a prominent role (51–53). MRI studies found trait anxiety being related to structural grey matter volume alterations as well as abnormal cortical thicknesses in limbic regions, such as the amygdala, parahippocampal gyrus, inferior temporal gyrus, and inferior frontal cortex (54, 55). Neuroimaging studies on state anxiety are scarce and their results are mostly regarded as the effect of anxious feelings induced by the MRI scanning procedure (37, 41). A potential difference upon comparing *T. gondii* -infected with uninfected ADHD individuals has not been investigated yet using MRI analysis of state or trait anxiety.

### Impact of ADHD medication

In the ADHD group of our study, more than half of participants had been treated with ADHD medication (MPH, atomoxetine or lisdexamphetamine) prior to study participation. All MPH-medicated patients took long-acting (extended-release) MPH formulations, as these are the primary licensed options for adult individuals with ADHD in Germany, with immediate-release forms generally only approved for off-label use (56). Moreover, the study was performed at a time when lisdexamphetamine in Germany had been approved for off-label use. In neuroimaging studies, for example, a wash out phase of 24 to 48 hours based on pharmacokinetics is most commonly used to approximate a stimulant naïve state (57). Although a recent study on wash-out status in functional connectivity revealed no significant differences in functional connectivity between stimulant washout groups (on, off, and washout) (57), previous studies have shown an increase in the amount of available dopamine in the brain, even following washout, and in individuals who were previously on psychostimulants (58). This suggests that long-term psychostimulant use can contribute to a lasting effect on the brain beyond a standard washout period (57). In our study, all participants underwent full diagnostic process prior to study begin, comprising of diagnostic interviews as well as self-rating scales in German. The diagnosis of ADHD in adulthood and other psychiatric disorders were established by psychiatric experts and dosage of ADHD medication had been reduced stepwise over several weeks until a minimal dosage was reached, which could finally be terminated three days prior to study participation. Regarding this discontinuation procedure, which was monitored by the treating physicians of the outpatient clinic, no clinical withdrawal effects were observed. However, as the majority of ADHD-patients in our study had been medicated with long-acting methylphenidate prior to study participation, a 3-day washout may be insufficient for complete pharmacological clearance. Therefore, pharmacological ADHD treatment was included as confounder in the multiple linear regression analyses regarding the dependent variables “leukocyte count”, “state anxiety” and “trait anxiety”. Adjusted and corrected for confounders, pharmacological ADHD treatment was not associated with the investigated dependent variables of our study. Nevertheless, despite of carefully performed drug reduction, symptom assessments through experts, and statistical adjustments, it cannot be barred that individual baseline anxiety levels might have been altered artificially due to medication cessation effects arising from medication withdrawal. Given the still insufficiently investigated impact of methylphenidate on the immune system with observed changes in cellular and humoral immunity (59, 60), the results of the study have to be judged with care and the presented limitations taken into account in future studies.

### Gender-specific differences

One study from Iran, conducted with healthy students, that completed a “General Health Questionnaire”, found *T. gondii* infected women having significantly lower scores in anxiety/insomnia as compared with non-infected women, while no significant differences between infected and non-infected men were found (61). In contrast to our study, the participants did not have any major psychiatric disorder, neurological disease or major physical disorder (61). However, our analyses of the ADHD group revealed, although without statistical significance, an association between gender and trait anxiety in seropositive individuals. This may be due to the limited number of seropositive individuals and should be reevaluated in a larger study sample. Gender-specific differences in our results and prior published data indicate that a reduction of anxiety may also be linked to the hormonal system.

Increased levels of circulating testosterone induce an epigenetic change in the DNA methylation of the arginine vasopressin promoter, which leads to a greater expression of arginine vasopressin in the medial amygdala (62, 63). It is discussed that affecting the medial amygdala vasopressin system reduces fear in *T. gondii* infected rodents (64). Lost fear in infected animals could also be demonstrated by systemically inducing the epigenetic change in DNA methylation of the arginine vasopressin promoter in the medial amygdala (62), and investigations in *T. gondii* infected rodents even suggest an influence of the parasite on the testosterone level in infected hosts (65). However, whether the suspected ability of *T. gondii* to modify the amygdala function in rodents may be extended to humans, remains hypothetical, and the impact of sex hormones in humans with latent *T. gondii* infection remains to be further investigated in future studies (66).

### Immunological considerations

Latent infections with *T. gondii* in humans have shown to be associated with elevated biomarkers of chronic inflammation (67) and growing evidence indicates that inflammation is involved in various psychiatric disorders, including ADHD (8, 68, 69).

Especially, inflammatory biomarkers including leukocytes, CRP, and IgG have recently been shown to be associated with a subsequent risk of psychiatric disorders (70).

With regard to *T. gondii*, it is known that, despite a strong immune response to the parasite, the chronic infection may persist in the host (71). The immune system is a complex set of physiological mechanisms and aims to defend the body against infectious agents (e.g. bacteria, viruses, parasites, fungi) to prevent them from causing diseases (19). The main biological responses comprise of an acute response component (e.g. CRP, white blood cells as leukocytes, IgM) and adaptive response component (e.g. IgG). In our study, *T. gondii* seropositivity, the CRP level, and the number of smoked cigarettes had a significant influence on the leukocyte count. CRP and white blood cells are known to correlate with each other (19). Also, previous studies demonstrated that leukocyte counts were higher in smokers of a large number of cigarettes (72). A recently published review discussed numerous findings regarding host defenses against *T. gondii* and the counter-defense mechanisms of the parasite in animals and humans (73). Although it is well known that various factors released from *T. gondii* infected cells strongly affect the subsequent activation of immune responses, the immunological responses described have been mainly studied using gene knockout mice (73). Thus, the molecular mechanisms of immune response in the human body against *T. gondii* has not yet been fully elucidated (73). Moreover, *T. gondii* seropositivity in humans reflects prior exposure and does not necessarily confirm chronic infection with functionally relevant tissue cysts. With regard to trait anxiety symptoms in ADHD patients, the results of our study indicate that acute response components (leukocytes) and adaptive response components of the immune system (IgG - serointensity) may play a more important role than the age of *T. gondii* seropositivity in the body of our study participants, represented by strength of IgG-avidity. In particular, the presented study illustrates a lower trait anxiety in ADHD patients correlating with the serum levels of anti-*T. gondii* IgG but not with IgG-avidity. While previous findings suggest an influence of the age of *T. gondii* infections on the core symptom severity in ADHD patients, measured via the avidity index of anti-*T. gondii* IgG (14), an effect of the avidity index could not be demonstrated in our presented study. In summary, this study is the first to show an association between *T. gondii* serointensity and trait anxiety in ADHD patients. Our results are in line with several studies on *T. gondii*, highlighting the importance of serointensity with regard to several psychiatric diseases (3, 74).

A recently published cohort study reported that, corresponding to the anti-inflammatory role of IgG, a higher level of IgG in general was associated with a lower risk of psychiatric disorders (70). In our study population, higher levels of anti-*T. gondii* IgG were significantly associated with lower trait anxiety scores. This result suggests that the effect of *T. gondii* on anxiety might depend on the individual immunological response. However, the underlying mechanisms for the associations of psychiatry disorders with biomarkers such as leukocytes and IgG still remain unknown (70). Impairments of neurotransmission, microglia activation, or blood brain barrier disruption are some of the potential explanations currently discussed in the literature (70) and need to be investigated in future studies.

There is increasing evidence that different serotypes of *T. gondii* may confer different clinically relevant outcomes, as an increased impairment and inflammation was reported depending on the serotypes of *T. gondii* strains in mice (75). Results of further studies indicate that the cyst load in the brain of infected mice might be associated with the severity of *T. gondii*-induced behavioral alterations (76). As consequence, this disregarded confounding factor need to be evaluated in future investigations.

We acknowledge the limited specificity of the included inflammatory and psychological markers in our study, as their non-specific nature and susceptibility to multiple confounders limit their interpretability as indicators of *T. gondii*-related processes. In future studies, more specific biomarkers should be included to allow further interpretations. Furthermore, the presented results and discussed neurobiological mechanisms should be judged with care as the underlying research derived almost exclusively from rodent and other mammal models and has been extrapolated to humans. Our interpretation is of speculative nature as human-specific evidence of the discussed mechanisms of behavioral or psychiatric effects attributable to *T. gondii* infection is lacking.

### Seroprevalence of *T. gondii*

It is well established, that the seroprevalence of *T. gondii* in humans varies between 1 and 100% and is depending, among others, on geographic factors, hygiene standards, eating habits, and age (12, 32, 77–83). For Germany, current data show a high rate of *T. gondii* seroprevalence, ranging from 20% to 77% depending on age, with a 95% confidence interval for individuals aged 18 to 29 years [17.0; 23.1] and for those aged 70 to 79 years [72.7; 80.5] (84, 85). In line with these national data, we have previously published the seroprevalence of *T. gondii* of the study cohort, which was also conducted in Germany and at 20% in a study population of 31.8 ± 10.4 years in mean (14). In humans, ingesting the parasite’s cysts through cyst-carrying undercooked meat, oocyst-contaminated soil, or contact with fecal material of infected cats have been found as the main risk factors of infection with *T. gondii* and a subsequent seropositivity (84). Data regarding risk factors of infection of the investigated study cohort have been published previously, indicating individuals with ADHD compared to controls to have more contact with cats, lower educational status, and to live more often in towns than in cities (14). Furthermore, even, if not statistically significant, individuals with ADHD tended to have more direct soil contact without wearing gloves (14), probably because of inattentive or impulsive behavior. Regarding investigated eating habits, patient and control groups were well balanced with regard to raw/undercooked meat consumption. With regard to marital status, the proportions of participants who had never been married were balanced between ADHD and controls (14). It had been assumed that ADHD-related symptoms, like inattention and impulsivity, may contribute to increased risk of infection with *T. gondii* through different pathways of infection. It has also been hypothesized that, for example, impulsivity may lead to reduced hygiene practices or greater contact with cats, the primary source of *T. gondii* oocysts. Moreover, inattention may result in less careful food handling, such as consuming undercooked meat. The statistics reveal that male sex, age, never been married, and raw/undercooked meat consumption were found predictive for seropositivity of *T. gondii* in the ADHD group (14). Other examined and less-fitting risk factors of infection did not reach statistical significance. With regard to the entire study sample, the same risk factors had been found to be associated with *T. gondii* seropositivity. As the study sample was well balanced by age and sex, and both groups were balanced with regard to the predictive risk factors of infection, our study did not reveal an ADHD-related plausibly increased exposure to *T. gondii* regarding the assessed hygiene standards or behavioral risk factors of infection in individuals with ADHD. Moreover, those previously published data is in line with population-based investigations on *T. gondii* seroprevalence (84). However, additional risk factors (e.g. cleaning hands, washing vegetables, contact with cat litter, et cetera) and larger sample sizes should be considered in future studies.

### Risk-taking behavior and anxiety

Anxiety, in general, can be regarded as a dynamic emotion that helps to avoid potential threats, and trait anxiety, in particular, has been shown to be associated with risk decision making (86). In concordance, results of a recent MRI study revealed neuroanatomical and functional differences between state and trait anxiety demonstrating functional changes in the default mode network (DMN) and salience network (SN), which may lead to changes in the perception of danger (37). Underestimation of danger may lead to risk-taking behaviors, defined as actions with a high-probability of undesirable outcomes endangering personal health and well-being (87). ADHD patients are often prone to risk-taking behavior resulting in higher rates of accidents, injuries, and mortality by deaths from unnatural causes, especially accidents (87–89). Concurrently, several studies reported a positive correlation between traffic accidents and the prevalence of *T. gondii* (12). Given the results of the presented study, reduction of trait anxiety in individuals with ADHD through *T. gondii* seropositivity and serointensity could contribute to aggravation of risk-taking and dangerous behaviors (e.g., reckless driving, substance use). In this circumstance, *T. gondii* may impose an additional risk in individuals with present ADHD. Therefore, future studies exploring the impact of *T. gondii* and ADHD on anxiety-dependent risk-taking behavior are required. While high trait anxiety in ADHD has been associated with negative clinical aspects like higher functional impairments or the need for adjunctive pharmacological treatments (38, 42, 90), the clinical impact of a reduced trait anxiety in ADHD, as observed in the presented study, needs to be further investigated. In the context of ADHD, an anti-*T. gondii* IgG dependent reduced trait anxiety could display an additional risk factor for patients due to an increased risk affinity, which might impact ADHD severity and treatment.

Altogether, our results do not aim to provide neurobiological evidence but to point at the underestimated relevance of *T. gondii* on the clinical course of ADHD patients. Our findings assume that *T. gondii* may be a so far highly disregarded clinical influencing factor in ADHD, Especially in terms of anxiety, risk-taking behavior, and risk affinity. Further research is necessary to clarify whether diagnostic implications for *T. gondii* in ADHD can be derived.

However, longitudinal study designs will be necessary to verify if *T. gondii* seropositivity or serointensity can predict a decline in anxiety in ADHD. As it comes to the question of behavioral changes, studies should include longitudinal observations of individual hosts before and after infection (9).

### Further limitations

This study is a case-control study and is not population-based. Moreover, individual immunity, resistance of the host, severity and timing of infection, epigenetic as well as confounding medical and environmental factors are important additional aspects that are difficult to assess, but need to be taken into account when discussing the clinical relevance of our findings (91). With regard to the questionnaire used in this study, it has also to be acknowledged that the STAI appears efficient in distinguishing between clinical and control samples; however, due to comorbidities symptom overlap, some items may be falsely elevated (36).

Moreover, despite of the high *T. gondii* seroprevalence in our study population, the results should be judged carefully, given the small total number of seropositive ADHD patients and the small sample sizes in various categories of the analyses.

## Conclusion

Our data is the first revealing an influence of *T. gondii* seropositivity on trait anxiety in a serointensity-dependent manner in individuals with ADHD. No association was found between *T. gondii* and anxiety disorders. With regard to our clinical findings, additional studies are mandatory to clarify the underlying neurobiological mechanisms. Further research is needed to evaluate the clinical impact of reduced trait anxiety for seropositive ADHD patients and possible implications for diagnostic and treatment.

## Data Availability

The raw data supporting the conclusions of this article will be made available by the authors, without undue reservation.

## Supplementary material

Supplementary Table 1A Multiple linear regression model of *T. gondii* seropositivity and trait anxiety in ADHD cases. B, unstandardized coefficient; β, standardized coefficient; *p*, p-value; CI, 95% confidence interval for [B]; *T. gondii*, *Toxoplasma gondii*; ADHD, attention-deficit/hyperactivity disorder; BPD, borderline personality disorder; bold, significant result (*p* < 0.05). “No” is the reference category for all categorical variables.

Supplementary Table 1B Multiple linear regression model of *T. gondii* seropositivity and state anxiety in ADHD cases. B, unstandardized coefficient; β, standardized coefficient; *p*, p-value; CI, 95% confidence interval for [B]; *T. gondii*, *Toxoplasma gondii*; ADHD, attention-deficit/hyperactivity disorder; BPD, borderline personality disorder; bold, significant result (*p* < 0.05). “No” is the reference category for all categorical variables.

Supplementary Table 2A Multiple linear regression model of serointensity and trait anxiety in ADHD cases. B, unstandardized coefficient; β, standardized coefficient; *p*, p-value; CI, 95% confidence interval for [B]; IgG, anti-*T. gondii* immunoglobulin G; ADHD, attention-deficit/hyperactivity disorder; BPD, borderline personality disorder; bold, significant result (*p* < 0.05). “No” is the reference category for all categorical variables.

Supplementary Table 2B Multiple linear regression model of serointensity and state anxiety in ADHD cases.

## References

[1] Berenreiterova M Flegr J Kubena AA Nemec P . The distribution of Toxoplasma gondii cysts in the brain of a mouse with latent toxoplasmosis: implications for the behavioral manipulation hypothesis. PloS One. (2011) 6:e28925. doi: 10.1371/journal.pone.0028925, PMID: 22194951 PMC3237564

[2] Berdoy M Webster JP Macdonald DW . Fatal attraction in rats infected with Toxoplasma gondii. Proc Biol Sci. (2000) 267:1591–4. doi: 10.1098/rspb.2000.1182, PMID: 11007336 PMC1690701

[3] Sutterland AL Fond G Kuin A Koeter MW Lutter R van Gool T . Beyond the association. Toxoplasma gondii in schizophrenia, bipolar disorder, and addiction: systematic review and meta-analysis. Acta Psychiatr Scand. (2015) 132:161–79. doi: 10.1111/acps.12423, PMID: 25877655

[4] Milne G Webster JP Walker M . Toxoplasma gondii: anUnderestimated threat? Trends Parasitol. (2020) 36:959–69. doi: 10.1016/j.pt.2020.08.005, PMID: 33012669

[5] Virus MA Ehrhorn EG Lui LM Davis PH . Neurological and neurobehavioral disorders associated with toxoplasma gondii infection in humans. J Parasitol Res. (2021) 2021:6634807. doi: 10.1155/2021/6634807, PMID: 34712493 PMC8548174

[6] Flegr J Horáček J . Negative effects of latent toxoplasmosis on mental health. Front Psychiatry. (2019) 10:1012. doi: 10.3389/fpsyt.2019.01012, PMID: 32132937 PMC7040223

[7] Kohler-Forsberg O Petersen L Gasse C Mortensen PB Dalsgaard S Yolken RH . A nationwide study in Denmark of the association between treated infections and the subsequent risk of treated mental disorders in children and adolescents. JAMA Psychiatry. (2018) 76:271–9. doi: 10.1001/jamapsychiatry.2018.3428, PMID: 30516814 PMC6439826

[8] Dunn G Nigg J Sullivan E . Neuroinflammation as a risk factor for attention deficit hyperactivity disorder. Pharmacol Biochem Behavior. (2019) 182:22–34. doi: 10.1016/j.pbb.2019.05.005, PMID: 31103523 PMC6855401

[9] Worth AR Andrew Thompson RC Lymbery AJ . Reevaluating the evidence for Toxoplasma gondii-induced behavioural changes in rodents. Adv parasitology. (2014) 85:109–42. doi: 10.1016/B978-0-12-800182-0.00003-9, PMID: 24928181

[10] Bay-Richter C Petersen E Liebenberg N Elfving B Wegener G . Latent toxoplasmosis aggravates anxiety- and depressive-like behaviour and suggest a role of gene-environment interactions in the behavioural response to the parasite. Behav Brain Res. (2019) 364:133–9. doi: 10.1016/j.bbr.2019.02.018, PMID: 30768994

[11] Gonzalez LE Rojnik B Urrea F Urdaneta H Petrosino P Colasante C . Toxoplasma gondii infection lower anxiety as measured in the plus-maze and social interaction tests in rats A behavioral analysis. Behav Brain Res. (2007) 177:70–9. doi: 10.1016/j.bbr.2006.11.012, PMID: 17169442

[12] Flegr J Prandota J Sovickova M Israili ZH . Toxoplasmosis--a global threat. Correlation of latent toxoplasmosis with specific disease burden in a set of 88 countries. PloS One. (2014) 9:e90203. doi: 10.1371/journal.pone.0090203, PMID: 24662942 PMC3963851

[13] Coccaro EF Lee R Groer MW Can A Coussons-Read M Postolache TT . Toxoplasma gondii infection: relationship with aggression in psychiatric subjects. J Clin Psychiatry. (2016) 77:334–41. doi: 10.4088/JCP.14m09621, PMID: 27046307

[14] Lam AP de Sordi D Müller HHO Lam MC Carl A Kohse KP . Aggravation of symptom severity in adult attention-deficit/hyperactivity disorder by latent Toxoplasma gondii infection: a case–control study. Sci Rep. (2020) 10:14382. doi: 10.1038/s41598-020-71084-w, PMID: 32873854 PMC7463265

[15] Fayyad J Sampson NA Hwang I Adamowski T Aguilar-Gaxiola S Al-Hamzawi A . The descriptive epidemiology of DSM-IV Adult ADHD in the World Health Organization World Mental Health Surveys. Attention deficit hyperactivity Disord. (2017) 9:47–65. doi: 10.1007/s12402-016-0208-3, PMID: 27866355 PMC5325787

[16] Biederman J . Impact of comorbidity in adults with attention-deficit/hyperactivity disorder. J Clin Psychiatry. (2004) 65 Suppl 3:3–7. 15046528

[17] Katzman MA Bilkey TS Chokka PR Fallu A Klassen LJ . Adult ADHD and comorbid disorders: clinical implications of a dimensional approach. BMC Psychiatry. (2017) 17:302–. doi: 10.1186/s12888-017-1463-3, PMID: 28830387 PMC5567978

[18] Libutzki B Ludwig S May M Jacobsen RH Reif A Hartman CA . Direct medical costs of ADHD and its comorbid conditions on basis of a claims data analysis. Eur psychiatry: J Assoc Eur Psychiatrists. (2019) 58:38–44. doi: 10.1016/j.eurpsy.2019.01.019, PMID: 30802682

[19] Santaolalla A Sollie S Rislan A Josephs DH Hammar N Walldius G . Association between serum markers of the humoral immune system and inflammation in the Swedish AMORIS study. BMC Immunol. (2021) 22:61. doi: 10.1186/s12865-021-00448-2, PMID: 34488637 PMC8420021

[20] Retz-Junginger P Retz W Blocher D Stieglitz RD Georg T Supprian T . Reliability and validity of the Wender-Utah-Rating-Scale short form. Retrospective assessment of symptoms for attention deficit/hyperactivity disorder. Der Nervenarzt. (2003) 74:987–93. doi: 10.1007/s00115-002-1447-4, PMID: 14598035

[21] Rosler M Retz W Retz-Junginger P Thome J Supprian T Nissen T . Tools for the diagnosis of attention-deficit/hyperactivity disorder in adults. Self-rating Behav questionnaire Diagn checklist. Der Nervenarzt. (2004) 75:888–95. doi: 10.1007/s00115-003-1622-2, PMID: 15378249

[22] Conners CK Erhardt D Sparrow EP . Conners’ adult ADHD rating scales, technical manual. New York: Multi-Health Systems (1999).

[23] Christiansen H Kis B Hirsch O Philipsen A Henneck M Panczuk A . German validation of the Conners Adult ADHD Rating Scales-self-report (CAARS-S) I: factor structure and normative data. Eur psychiatry: J Assoc Eur Psychiatrists. (2011) 26:100–7. doi: 10.1016/j.eurpsy.2009.12.024, PMID: 20619613

[24] Christiansen H Hirsch O Philipsen A Oades RD Matthies S Hebebrand J . German validation of the conners adult ADHD rating scale-self-report: confirmation of factor structure in a large sample of participants with ADHD. J attention Disord. (2013) 17:690–8. doi: 10.1177/1087054711435680, PMID: 22441889

[25] Wittchen H-U Zaudig M Fydrich T . SKID–Strukturiertes Klinisches Interview für DSM IV. Achse I und II. Göttingen Hogrefe. (1997).

[26] Beck A Steer R Brown G . Beck depression inventory. San Antonio, TX: Psychological Corp (1996).

[27] Freitag CM Retz-Junginger P Retz W Seitz C Palmason H Meyer J . Evaluation der deutschen Version des Autismus-Spektrum-Quotienten (AQ) - die Kurzversion AQ-k. Z für Klinische Psychol und Psychotherapie. (2007) 36:280–9. doi: 10.1026/1616-3443.36.4.280, PMID: 33157507

[28] Spielberger CD Schaffner P Laux P . State-trait-angstinventar (STAI). BELTZTEST. (1983).

[29] Pignanelli S . Laboratory diagnosis of Toxoplasma gondii infection with direct and indirect diagnostic techniques. Indian J Pathol Microbiol. (2011) 54:786–9. doi: 10.4103/0377-4929.91503, PMID: 22234111

[30] Tonhajzerova I Ondrejka I Farsky I Visnovcova Z Mestanik M Javorka M . Attention deficit/hyperactivity disorder (ADHD) is associated with altered heart rate asymmetry. Physiol Res. (2014) 63 Suppl 4:S509–19. doi: 10.33549/physiolres.932919, PMID: 25669682

[31] Wigman JT van Os J Abidi L Huibers MJ Roelofs J Arntz A . Subclinical psychotic experiences and bipolar spectrum features in depression: association with outcome of psychotherapy. Psychol Med. (2014) 44:325–36. doi: 10.1017/S0033291713000871, PMID: 23651602

[32] Flegr J . Effects of toxoplasma on human behavior. Schizophr bulletin. (2007) 33:757–60. doi: 10.1093/schbul/sbl074, PMID: 17218612 PMC2526142

[33] Postolache TT Wadhawan A Rujescu D Hoisington AJ Dagdag A Baca-Garcia E . Toxoplasma gondii, suicidal behavior, and intermediate phenotypes for suicidal behavior. Front Psychiatry. (2021) 12:665682. doi: 10.3389/fpsyt.2021.665682, PMID: 34177652 PMC8226025

[34] Dowran B Khanaliha K Mohammadzadeh T . Toxoplasmosis and anxiety: A review study. Int J Enteric Pathog (2021) 9:83–9. doi: 10.34172/ijep.2021.17, PMID: 41255731

[35] Grenier S Payette MC Gunther B Askari S Desjardins FF Raymond B . Association of age and gender with anxiety disorders in older adults: A systematic review and meta-analysis. Int J Geriatr Psychiatry. (2019) 34:397–407. doi: 10.1002/gps.5035, PMID: 30444008

[36] Grogan K Gormley CI Rooney B Whelan R Kiiski H Naughton M . Differential diagnosis and comorbidity of ADHD and anxiety in adults. Br J Clin Psychol. (2018) 57:99–115. doi: 10.1111/bjc.12156, PMID: 28895146

[37] Saviola F Pappaianni E Monti A Grecucci A Jovicich J De Pisapia N . Trait and state anxiety are mapped differently in the human brain. Sci Rep. (2020) 10:11112. doi: 10.1038/s41598-020-68008-z, PMID: 32632158 PMC7338355

[38] Pehlivanidis A Papanikolaou K Spyropoulou AC Papadimitriou GN . Comorbid attention-deficit/hyperactivity disorder in adult psychiatric outpatients with depressive or anxiety disorders. Int J Psychiatry Clin practice. (2014) 18:265–71. doi: 10.3109/13651501.2014.941878, PMID: 24998681

[39] Müller BW Gimbel K Keller-Pliessnig A Sartory G Gastpar M Davids E . Neuropsychological assessment of adult patients with attention-deficit/hyperactivity disorder. Eur Arch Psychiatry Clin Neurosci. (2007) 257:112–9. doi: 10.1007/s00406-006-0688-9, PMID: 17200879

[40] Kordahji H Ben-David S Elkana O . Attachment anxiety moderates the association between ADHD and psychological distress. Psychiatr Q. (2021) 92:1711–24. doi: 10.1007/s11126-021-09919-6, PMID: 34245401

[41] González-Castro P Rodríguez C Cueli M García T Alvarez-García D . State, trait anxiety and selective attention differences in Attention Deficit Hyperactivity Disorder (ADHD) subtypes. Int J Clin Health Psychol. (2015) 15:105–12. doi: 10.1016/j.ijchp.2014.10.003, PMID: 30487827 PMC6224773

[42] Oh Y Yoon HJ Kim JH Joung YS . Trait anxiety as a mediator of the association between attention deficit hyperactivity disorder symptom severity and functional impairment. Clin Psychopharmacol neuroscience: Off Sci J Korean Coll Neuropsychopharmacol. (2018) 16:407–14. doi: 10.9758/cpn.2018.16.4.407, PMID: 30466213 PMC6245288

[43] Areces D Rodríguez C García T Cueli M González-Castro P . The influence of state and trait anxiety on the achievement of a virtual reality continuous performance test in children and adolescents with ADHD symptoms. J Clin Med. (2021) 10:2534. doi: 10.3390/jcm10122534, PMID: 34200987 PMC8229147

[44] Mitra R Sapolsky RM Vyas A . Toxoplasma gondii infection induces dendritic retraction in basolateral amygdala accompanied by reduced corticosterone secretion. Dis Models mechanisms. (2013) 6:516–20. doi: 10.1242/dmm.009928, PMID: 23104989 PMC3597033

[45] Tabaie EZ Gao Z Kachour N Ulu A Gomez S Figueroa ZA . Toxoplasma gondii infection of neurons alters the production and content of extracellular vesicles directing astrocyte phenotype and contributing to the loss of GLT-1 in the infected brain. PloS Pathog. (2025) 21:e1012733. doi: 10.1371/journal.ppat.1012733, PMID: 40523037 PMC12193631

[46] Kaushik M Lamberton PH Webster JP . The role of parasites and pathogens in influencing generalised anxiety and predation-related fear in the mammalian central nervous system. Horm Behav. (2012) 62:191–201. doi: 10.1016/j.yhbeh.2012.04.002, PMID: 22521209

[47] Vyas A Kim SK Sapolsky RM . The effects of toxoplasma infection on rodent behavior are dependent on dose of the stimulus. Neuroscience. (2007) 148:342–8. doi: 10.1016/j.neuroscience.2007.06.021, PMID: 17683872 PMC2430144

[48] Markovitz AA Simanek AM Yolken RH Galea S Koenen KC Chen S . Toxoplasma gondii and anxiety disorders in a community-based sample. Brain behavior immunity. (2015) 43:192–7. doi: 10.1016/j.bbi.2014.08.001, PMID: 25124709

[49] Weger M Sandi C . High anxiety trait: A vulnerable phenotype for stress-induced depression. Neurosci Biobehav Rev. (2018) 87:27–37. doi: 10.1016/j.neubiorev.2018.01.012, PMID: 29407523

[50] Martin EI Ressler KJ Binder E Nemeroff CB . The neurobiology of anxiety disorders: brain imaging, genetics, and psychoneuroendocrinology. Psychiatr Clin North Am. (2009) 32:549–75. doi: 10.1016/j.psc.2009.05.004, PMID: 19716990 PMC3684250

[51] Etkin A Klemenhagen KC Dudman JT Rogan MT Hen R Kandel ER . Individual differences in trait anxiety predict the response of the basolateral amygdala to unconsciously processed fearful faces. Neuron. (2004) 44:1043–55. doi: 10.1016/j.neuron.2004.12.006, PMID: 15603746

[52] Bishop SJ Duncan J Lawrence AD . State anxiety modulation of the amygdala response to unattended threat-related stimuli. J Neurosci. (2004) 24:10364–8. doi: 10.1523/JNEUROSCI.2550-04.2004, PMID: 15548650 PMC6730310

[53] Derryberry D Reed MA . Anxiety-related attentional biases and their regulation by attentional control. J Abnorm Psychol. (2002) 111:225–36. doi: 10.1037/0021-843X.111.2.225, PMID: 12003445

[54] Hu Y Dolcos S . Trait anxiety mediates the link between inferior frontal cortex volume and negative affective bias in healthy adults. Soc Cognit Affect Neurosci. (2017) 12:775–82. doi: 10.1093/scan/nsx008, PMID: 28158829 PMC5460040

[55] Potvin O Catheline G Bernard C Meillon C Bergua V Allard M . Gray matter characteristics associated with trait anxiety in older adults are moderated by depression. Int psychogeriatrics. (2015) 27:1813–24. doi: 10.1017/S1041610215000836, PMID: 26059837

[56] Abdel-Hamid M Lyons N Specka M Bartels C Belz M Hessmann P . Diversion and abuse of prescribed methylphenidate – A survey of an outpatient clinic for adult persons with ADHD. Pharmacopsychiatry. (2025) 58:198–9. doi: 10.1055/a-2545-1286, PMID: 40148130

[57] Harkness K Bray S Murias K . The role of stimulant washout status in functional connectivity of default mode and fronto-parietal networks in children with neurodevelopmental conditions. Res Dev disabilities. (2024) 146:104691. doi: 10.1016/j.ridd.2024.104691, PMID: 38340416

[58] Fusar-Poli P Rubia K Rossi G Sartori G Balottin U . Striatal dopamine transporter alterations in ADHD: pathophysiology or adaptation to psychostimulants? A meta-analysis. Am J Psychiatry. (2012) 169:264–72. doi: 10.1176/appi.ajp.2011.11060940, PMID: 22294258

[59] Auci DL Fikrig S Rodriquez J . Methylphenidate and the immune system. J Am Acad Child Adolesc Psychiatry. (1997) 36:1015–6. doi: 10.1097/00004583-199708000-00004, PMID: 9256577

[60] Motaghinejad M Motevalian M Shabab B Fatima S . Effects of acute doses of methylphenidate on inflammation and oxidative stress in isolated hippocampus and cerebral cortex of adult rats. J Neural Transm (Vienna). (2017) 124:121–31. doi: 10.1007/s00702-016-1623-5, PMID: 27682635

[61] Khademvatan S Izadi-Mazidi M Saki J Khajeddin N . Mental health and latent toxoplasmosis: comparison of individuals with and without anti-toxoplasma antibodies. World Health population. (2016) 17:39–46. doi: 10.12927/whp.2016.24938, PMID: 28332976

[62] Hari Dass SA Vyas A . Toxoplasma gondii infection reduces predator aversion in rats through epigenetic modulation in the host medial amygdala. Mol ecology. (2014) 23:6114–22. doi: 10.1111/mec.12888, PMID: 25142402

[63] Tong WH Abdulai-Saiku S Vyas A . Testosterone reduces fear and causes drastic hypomethylation of arginine vasopressin promoter in medial extended amygdala of male mice. Front Behav Neurosci. (2019) 13:33. doi: 10.3389/fnbeh.2019.00033, PMID: 30863290 PMC6399424

[64] House PK Vyas A Sapolsky R . Predator cat odors activate sexual arousal pathways in brains of Toxoplasma gondii infected rats. PloS One. (2011) 6:e23277. doi: 10.1371/journal.pone.0023277, PMID: 21858053 PMC3157360

[65] Singh DK Hari Dass SA Abdulai-Saiku S Vyas A . Testosterone acts within the medial amygdala of rats to reduce innate fear to predator odor akin to the effects of toxoplasma gondii infection. Front Psychiatry. (2020) 11:630–. doi: 10.3389/fpsyt.2020.00630, PMID: 32714222 PMC7343892

[66] Lim A Kumar V Hari Dass SA Vyas A . Toxoplasma gondii infection enhances testicular steroidogenesis in rats. Mol ecology. (2013) 22:102–10. doi: 10.1111/mec.12042, PMID: 23190313

[67] Egorov AI Converse RR Griffin SM Styles JN Sams E Hudgens E . Latent Toxoplasma gondii infections are associated with elevated biomarkers of inflammation and vascular injury. BMC Infect Diseases. (2021) 21:188. doi: 10.1186/s12879-021-05882-6, PMID: 33602170 PMC7890825

[68] Yuan N Chen Y Xia Y Dai J Liu C . Inflammation-related biomarkers in major psychiatric disorders: a cross-disorder assessment of reproducibility and specificity in 43 meta-analyses. Transl Psychiatry. (2019) 9:233. doi: 10.1038/s41398-019-0570-y, PMID: 31534116 PMC6751188

[69] Felger JC . Imaging the role of inflammation in mood and anxiety-related disorders. Curr neuropharmacology. (2018) 16:533–58. doi: 10.2174/1570159X15666171123201142, PMID: 29173175 PMC5997866

[70] Zeng Y Chourpiliadis C Hammar N Seitz C Valdimarsdóttir UA Fang F . Inflammatory biomarkers and risk of psychiatric disorders. JAMA Psychiatry. (2024) 81:1118–29. doi: 10.1001/jamapsychiatry.2024.2185, PMID: 39167384 PMC11339698

[71] Khan IA Moretto M . Immune responses to Toxoplasma gondii. Curr Opin Immunol. (2022) 77:102226. doi: 10.1016/j.coi.2022.102226, PMID: 35785567

[72] Petitti DB Kipp H . The leukocyte count: associations with intensity of smoking and persistence of effect after quitting. Am J Epidemiol. (1986) 123:89–95. doi: 10.1093/oxfordjournals.aje.a114227, PMID: 3940445

[73] Sasai M Yamamoto M . Innate, adaptive, and cell-autonomous immunity against Toxoplasma gondii infection. Exp Mol Med. (2019) 51:1–10. doi: 10.1038/s12276-019-0353-9, PMID: 31827072 PMC6906438

[74] Rensch P Postolache TT Dalkner N Stross T Constantine N Dagdag A . Toxoplasma gondii IgG serointensity and cognitive function in bipolar disorder. Int J Bipolar Disord. (2024) 12:31. doi: 10.1186/s40345-024-00353-8, PMID: 39179937 PMC11343948

[75] Evangelista FF de Laet Sant’Ana P Ferreira WC Ferreira TA Dos Santos ML de Souza AH . The Brazilian Toxoplasma gondii strain BRI caused greater inflammation and impairment in anxiogenic behavior in mice, which was reverted by rosuvastatin treatment. Parasitol Res. (2023) 123:64. doi: 10.1007/s00436-023-08038-4, PMID: 38117414

[76] Boillat M Hammoudi P-M Dogga SK Pagès S Goubran M Rodriguez I . Neuroinflammation-associated aspecific manipulation of mouse predator fear by toxoplasma gondii. Cell Rep. (2020) 30:320–34.e6. doi: 10.1016/j.celrep.2019.12.019, PMID: 31940479 PMC6963786

[77] Akanmu AS Osunkalu VO Ofomah JN Olowoselu FO . Pattern of demographic risk factors in the seroprevalence of anti-Toxoplasma gondii antibodies in HIV infected patients at the Lagos University Teaching Hospital. Nigerian Q J Hosp Med. (2010) 20:1–4. doi: 10.4314/nqjhm.v20i1.57974, PMID: 20450022

[78] Montoya JG Liesenfeld O . Toxoplasmosis. Lancet (London England). (2004) 363:1965–76. doi: 10.1016/S0140-6736(04)16412-X, PMID: 15194258

[79] Lindova J Kubena AA Sturcova H Krivohlava R Novotna M Rubesova A . Pattern of money allocation in experimental games supports the stress hypothesis of gender differences in Toxoplasma gondii-induced behavioural changes. Folia parasitologica. (2010) 57:136–42. doi: 10.14411/fp.2010.017 20608476

[80] Lindova J Novotna M Havlicek J Jozifkova E Skallova A Kolbekova P . Gender differences in behavioural changes induced by latent toxoplasmosis. Int J Parasitol. (2006) 36:1485–92. doi: 10.1016/j.ijpara.2006.07.008, PMID: 16978630

[81] Furtado JM Smith JR Belfort R Jr. Gattey D Winthrop KL . Toxoplasmosis: a global threat. J Glob Infect Dis. (2011) 3:281–4. doi: 10.4103/0974-777X.83536, PMID: 21887062 PMC3162817

[82] Dubey JP . Advances in the life cycle of Toxoplasma gondii. Int J Parasitol. (1998) 28:1019–24. doi: 10.1016/S0020-7519(98)00023-X, PMID: 9724872

[83] Tenter AM Heckeroth AR Weiss LM . Toxoplasma gondii: from animals to humans. Int J Parasitol. (2000) 30:1217–58. doi: 10.1016/S0020-7519(00)00124-7, PMID: 11113252 PMC3109627

[84] Wilking H Thamm M Stark K Aebischer T Seeber F . Prevalence, incidence estimations, and risk factors of Toxoplasma gondii infection in Germany: a representative, cross-sectional, serological study. Sci Rep. (2016) 6:22551. doi: 10.1038/srep22551, PMID: 26936108 PMC4776094

[85] Pleyer U Gross U Schlüter D Wilking H Seeber F . Toxoplasmosis in Germany. Dtsch Arztebl Int. (2019) 116:435–44. doi: 10.3238/arztebl.2019.0435, PMID: 31423982 PMC6706837

[86] Xu P Gu R Broster LS Wu R Van Dam NT Jiang Y . Neural basis of emotional decision making in trait anxiety. J Neurosci. (2013) 33:18641–53. doi: 10.1523/JNEUROSCI.1253-13.2013, PMID: 24259585 PMC3834062

[87] Chen Y Liu P Yi S Fan C Zhao W Liu J . Investigating the shared genetic architecture between attention-deficit/hyperactivity disorder and risk taking behavior: A large-scale genomewide cross-trait analysis. J Affect Disord. (2024) 356:22–31. doi: 10.1016/j.jad.2024.03.107, PMID: 38565336

[88] Dalsgaard S Østergaard SD Leckman JF Mortensen PB Pedersen MG . Mortality in children, adolescents, and adults with attention deficit hyperactivity disorder: a nationwide cohort study. Lancet (London England). (2015) 385:2190–6. doi: 10.1016/S0140-6736(14)61684-6, PMID: 25726514

[89] Libutzki B Neukirch B Kittel-Schneider S Reif A Hartman CA . Risk of accidents and unintentional injuries in men and women with attention deficit hyperactivity disorder across the adult lifespan. Acta Psychiatr Scand. (2023) 147:145–54. doi: 10.1111/acps.13524, PMID: 36464800 PMC10107297

[90] Manoli A Liversedge SP Sonuga-Barke EJS Hadwin JA . The differential effect of anxiety and ADHD symptoms on inhibitory control and sustained attention for threat stimuli: A go/no-go eye-movement study. J attention Disord. (2020) 25:1919–30. doi: 10.1177/1087054720930809, PMID: 32513052 PMC8427811

[91] Carter CJ . Toxoplasmosis and polygenic disease susceptibility genes: extensive toxoplasma gondii host/pathogen interactome enrichment in nine psychiatric or neurological disorders. J Pathog. (2013) 2013:965046. doi: 10.1155/2013/965046, PMID: 23533776 PMC3603208

