# Supplementary material for: Toxoplasma gondii affects trait anxiety in adult ADHD
Source: Front Psychiatry. 2026 Mar 5;17:1766562. doi: 10.3389/fpsyt.2026.1766562 (PMC12999780; doi:10.3389/fpsyt.2026.1766562)
Supplement: Supplementary Table 1A — Multiple linear regression model of T. gondii seropositivity and trait anxiety in ADHD cases. B, unstandardized coefficient; β, standardized coefficient; p, p-value; CI, 95% confidence interval for [B]; T. gondii, Toxoplasma gondii; ADHD, attention-deficit/hyperactivity disorder; BPD, borderline personality disorder; bold, significant result (p < 0.05). “No” is the reference category for all categorical variables. [file Table1.pdf]

Supplementary Table 1a Multiple linear regression model of *T. gondii* seropositivity and trait anxiety in ADHD cases

Trait anxiety, ADHD cases							
**First Model: included variables (*n* = 70 , adjusted R^2 =0.226, *p* = 0.003)**							
						**95% CI**	
	**B**	**SE B**	***β***	**T**	***p***	**Lower**	**Upper**
**(Intercept)**	46.477	3.895		11.932	**0.000**	38.674	54.280
Age	0.063	0.109	0.072	0.577	0.566	-0.156	0.282
Sex	3.992	2.346	0.207	1.702	0.094	-0.707	8.691
T. gondii seropositivity	-6.265	2.403	-0.289	-2.607	**0.012**	-11.078	-1.451
BPD	9.671	3.409	0.402	2.837	**0.006**	2.842	16.500
Axis II disorder other than BPD	9.093	3.525	0.301	2.580	**0.013**	2.032	16.153
Affective disorder	3.908	2.332	0.202	1.676	0.099	-0.764	8.580
Anxiety disorder	1.710	2.860	0.071	0.598	0.552	-4.020	7.439
Substance abuse lifetime	-1.512	2.743	-0.070	-0.551	0.584	-7.006	3.982
Eating disorder	-3.872	5.576	-0.081	-0.694	0.490	-15.043	7.298
Neuroleptics	0.799	4.551	0.023	0.176	0.861	-8.317	9.915
Antidepressants	-0.002	2.962	0.000	-0.001	0.999	-5.935	5.931
Sedatives	1.063	6.369	0.022	0.167	0.868	-11.695	13.822
Current pharmacological ADHD treatment	-3.048	2.451	-0.158	-1.244	0.219	-7.959	1.862
**Final Model: included variables (*n* = 70, adjusted R^2 = 0.324, *p* < 0.001)**							
						**95% CI**	
	**B**	**SE B**	***β***	**T**	***p***	**Lower**	**Upper**
**(Intercept)**	46.580	1.829		25.465	**0.000**	42.926	50.234
Sex	3.889	2.027	0.202	1.918	0.060	-0.161	7.939
T. gondii seropositivity	-5.989	2.206	-0.277	-2.715	**0.009**	-10.396	-1.582
BPD	10.231	2.615	0.425	3.913	**0.000**	5.008	15.454
Axis II disorder other than BPD	10.016	3.146	0.331	3.183	**0.002**	3.731	16.302
Affective disorder	3.906	2.035	0.202	1.920	0.059	-0.159	7.971
**Final Model: excluded variables**							
			**β In**	**T**	***p***	**Partial Correlation**	**Collinearity Statistics Tolerance**
Antidepressants			0.005	0.043	0.966	0.005	0.706
Sedatives			0.001	0.005	0.996	0.001	0.852
Neuroleptics			0.002	0.021	0.983	0.003	0.744
Substance abuse lifetime			-0.02	-0.158	0.875	-0.020	0.849
Anxiety disorder			0.096	0.931	0.355	0.117	0.932
Eating disorder			-0.06	-0.596	0.553	-0.075	0.928
Age			0.072	0.674	0.503	0.085	0.855
Current pharmacological ADHD treatment			-0.1	-1.006	0.318	-0.126	0.928

B, unstandardized coefficient**;** β, standardized coefficient; *p*, p-value; CI, 95% confidence interval for [B]; *T. gondii*, *Toxoplasma gondii*; ADHD, attention-deficit/hyperactivity disorder; BPD, borderline personality disorder; bold, significant result (*p* < 0.05). “No” is the reference category for all categorical variables.

Supplementary Table 1b Multiple linear regression model of *T. gondii* seropositivity and state anxiety in ADHD cases

**State anxiety, ADHD cases**							
**First Model: included variables (*n* = 70 , adjusted R^2 = 0.117, *p* = 0.085)**							
						**95% CI**	
	**B**	**SE B**	***β***	**T**	***p***	**Lower**	**Upper**
**(Intercept)**	43.270	3.894		11.113	**0.000**	35.470	51.070
Age	0.166	0.109	0.209	1.516	0.135	-0.053	0.385
Sex	2.985	2.345	0.170	1.273	0.208	-1.712	7.682
T. gondii seropositivity	-4.060	2.402	-0.206	-1.691	0.096	-8.872	0.751
BPD	11.852	3.408	0.540	3.478	**0.001**	5.025	18.678
Axis II disorder other than BPD	9.511	3.523	0.345	2.699	**0.009**	2.453	16.569
Affective disorder	0.346	2.331	0.020	0.148	0.883	-4.324	5.016
Anxiety disorder	-4.688	2.859	-0.214	-1.640	0.107	-10.416	1.039
Substance abuse lifetime	-3.483	2.742	-0.177	-1.271	0.209	-8.975	2.009
Eating disorder	2.904	5.574	0.067	0.521	0.604	-8.262	14.071
Neuroleptics	-5.511	4.549	-0.176	-1.212	0.231	-14.624	3.601
Antidepressants	-0.303	2.961	-0.015	-0.102	0.919	-6.234	5.628
Sedatives	-3.846	6.367	-0.089	-0.604	0.548	-16.599	8.908
Current pharmacological ADHD treatment	-0.921	2.450	-0.052	-0.376	0.708	-5.830	3.988
**Final Model: included variables (*n* = 70, adjusted R^2 = 0.117, *p* = 0.006)**							
						**95% CI**	
	**B**	**SE B**	***β***	**T**	***p***	**Lower**	**Upper**
**(Intercept)**	47.548	1.198		39.691	**0.000**	45.157	49.939
BPD	6.880	2.521	0.314	2.729	**0.008**	1.848	11.912
Axis II disorder other than BPD	7.577	3.170	0.275	2.390	**0.020**	1.250	13.903
**Final Model: excluded variables**							
			**β In**	**T**	***p***	**Partial Correlation**	**Collinearity Statistics Tolerance**
Antidepressants			-0.028	-0.218	0.828	-0.027	0.800
Affective disorder			-0.035	-0.290	0.772	-0.036	0.892
Current pharmacological ADHD treatment			0.045	0.386	0.700	0.048	0.937
Eating disorder			0.012	0.105	0.917	0.013	0.991
Sedatives			-0.08	-0.670	0.505	-0.082	0.902
Substance abuse lifetime			-0.12	-1.025	0.309	-0.125	0.933
Sex			0.141	1.206	0.232	0.147	0.927
T. gondii seropositivity			-0.106	-0.931	0.355	-0.114	0.990
Age			0.125	1.055	0.295	0.129	0.916
Anxiety disorder			-0.123	-1.081	0.284	-0.132	0.986
Neuroleptics			-0.161	-1.274	0.207	-0.155	0.794

B, unstandardized coefficient**;** β, standardized coefficient; *p*, p-value; CI, 95% confidence interval for [B]; *T. gondii*, *Toxoplasma gondii*; ADHD, attention-deficit/hyperactivity disorder; BPD, borderline personality disorder; bold, significant result ($p < 0.05$). “No” is the reference category for all categorical variables.

Supplementary Table 2a Multiple linear regression model of serointensity and trait anxiety in ADHD cases

Trait anxiety, ADHD cases							
**First Model: included variables (*n* = 70 , adjusted R^2 = 0.25, *p* = 0.004)**							
						**95% CI**	
	**B**	**SE B**	***β***	**T**	***p***	**Lower**	**Upper**
**(Intercept)**	47.131	3.929		11.996	**0.000**	39.260	55.002
Age	0.047	0.110	0.054	0.426	0.672	-0.173	0.267
Sex	3.698	2.359	0.192	1.568	0.123	-1.028	8.423
IgG [U/ml]	-0.051	0.022	-0.259	-2.330	**0.023**	-0.094	-0.007
BPD	8.771	3.454	0.365	2.539	**0.014**	1.851	15.691
Axis II disorder other than BPD	7.855	3.554	0.260	2.210	**0.031**	0.735	14.976
Affective disorder	3.417	2.363	0.176	1.446	0.154	-1.317	8.152
Anxiety disorder	1.215	2.912	0.050	0.417	0.678	-4.618	7.048
Substance abuse lifetime	-1.695	2.777	-0.078	-0.610	0.544	-7.257	3.867
Eating disorder	-4.386	5.621	-0.092	-0.780	0.439	-15.646	6.874
Neuroleptics	1.060	4.600	0.031	0.230	0.819	-8.156	10.276
Antidepressants	0.295	3.011	0.014	0.098	0.922	-5.736	6.326
Sedatives	2.162	6.392	0.045	0.338	0.736	-10.643	14.966
Current pharmacological ADHD treatment	-2.942	2.477	-0.153	-1.188	0.240	-7.903	2.020
**Final Model: included variables (*n* = 70, adjusted R^2 = 0.313, *p* < 0.001)**							
						**95% CI**	
	**B**	**SE B**	***β***	**T**	***p***	**Lower**	**Upper**
**(Intercept)**	46.649	1.852		25.188	**0.000**	42.949	50.348
Sex	3.633	2.029	0.189	1.791	0.078	-0.420	7.687
IgG [U/ml]	-0.050	0.020	-0.258	-2.509	**0.015**	-0.091	-0.010
BPD	9.616	2.649	0.400	3.631	**0.001**	4.325	14.908
Axis II disorder other than BPD	9.003	3.157	0.298	2.851	**0.006**	2.695	15.310
Affective disorder	3.530	2.048	0.182	1.724	0.090	-0.561	7.621
**Final Model: excluded variables**							
			**β In**	**T**	***p***	**Partial Correlation**	**Collinearity Statistics Tolerance**
Antidepressants			0.02	0.166	0.868	0.021	0.699
Neuroleptics			0.024	0.208	0.836	0.026	0.751
Anxiety disorder			0.067	0.648	0.519	0.081	0.936
Sedatives			0.027	0.254	0.801	0.032	0.863
Age			0.056	0.516	0.607	0.065	0.869
Substance abuse lifetime			-0.022	-0.206	0.838	-0.026	0.846
Eating disorder			-0.065	-0.621	0.537	-0.078	0.929
Current pharmacological ADHD treatment			-0.095	-0.915	0.364	-0.114	0.928

B, unstandardized coefficient**;** β, standardized coefficient; *p*, p-value; CI, 95% confidence interval for [B]; IgG, anti-*T. gondii* immunoglobulin G ; ADHD, attention-deficit/hyperactivity disorder; BPD, borderline personality disorder; bold, significant result (*p* < 0.05). “No” is the reference category for all categorical variables.

Supplementary Table 2b Multiple linear regression model of serointensity and state anxiety in ADHD cases

State anxiety, ADHD cases							
**First Model: included variables (*n* = 70 , adjusted R^2 = 0.88, *p* = 0.143)**							
						**95% CI**	
	**B**	**SE B**	***β***	**T**	***p***	**Lower**	**Upper**
**(Intercept)**	43.705	3.949		11.068	**0.000**	35.794	51.615
Age	0.145	0.110	0.183	1.320	0.192	-0.075	0.366
Sex	2.618	2.371	0.149	1.104	0.274	-2.131	7.367
IgG [U/ml]	-0.021	0.022	-0.120	-0.975	0.334	-0.065	0.022
BPD	11.404	3.472	0.520	3.285	**0.002**	4.449	18.359
Axis II disorder other than BPD	8.806	3.572	0.319	2.465	**0.017**	1.649	15.962
Affective disorder	0.115	2.375	0.007	0.048	0.962	-4.643	4.874
Anxiety disorder	-4.815	2.926	-0.220	-1.645	0.106	-10.677	1.047
Substance abuse lifetime	-3.518	2.791	-0.178	-1.261	0.213	-9.108	2.072
Eating disorder	2.241	5.649	0.052	0.397	0.693	-9.076	13.558
Neuroleptics	-5.355	4.624	-0.171	-1.158	0.252	-14.618	3.907
Antidepressants	-0.317	3.026	-0.016	-0.105	0.917	-6.379	5.744
Sedatives	-2.780	6.424	-0.064	-0.433	0.667	-15.649	10.089
Current pharmacological ADHD treatment	-0.761	2.489	-0.043	-0.306	0.761	-5.748	4.226
**Final Model: included variables (*n* = 70, adjusted R^2 = 0.117, *p* = 0.006)**							
						**95% CI**	
	**B**	**SE B**	***β***	**T**	***p***	**Lower**	**Upper**
**(Intercept)**	47.548	1.198		39.691	**0.000**	45.157	49.939
BPD	6.880	2.521	0.314	2.729	**0.008**	1.848	11.912
Axis II disorder other than BPD	7.577	3.170	0.275	2.390	**0.020**	1.250	13.903
**Final Model: excluded variables**							
			**β In**	**T**	***p***	**Partial Correlation**	**Collinearity Statistics Tolerance**
Affective disorder			-0.035	-0.290	0.772	-0.036	0.892
Antidepressants			-0.028	-0.218	0.828	-0.027	0.800
Current pharmacological ADHD treatment			0.045	0.386	0.700	0.048	0.937
Eating disorder			0.012	0.105	0.917	0.013	0.991
Sedatives			-0.08	-0.670	0.505	-0.082	0.902
IgG [U/ml]			-0.035	-0.307	0.760	-0.038	0.973
Sex			0.141	1.206	0.232	0.147	0.927
Substance abuse lifetime			-0.12	-1.025	0.309	-0.125	0.933
Age			0.125	1.055	0.295	0.129	0.916
Anxiety disorder			-0.123	-1.081	0.284	-0.132	0.986
Neuroleptics			-0.161	-1.274	0.207	-0.155	0.794

B, unstandardized coefficient**;** β, standardized coefficient; *p*, p-value; CI, 95% confidence interval for [B]; IgG, anti-*T. gondii* immunoglobulin G ; ADHD, attention-deficit/hyperactivity disorder; BPD, borderline personality disorder; bold, significant result ($p < 0.05$). “No” is the reference category for all categorical variables.
